# Automated Detection and Ordinal Severity Grading of Periapical Lesions on Panoramic Radiographs Using a Lesion-Preserving Tiling and Ordinal-Aware YOLO Framework

**DOI:** 10.3390/diagnostics16172778

**Published:** 2026-08-29

**Authors:** Faruk Oztekin, Oguzhan Katar, Nurefsan Avci, Furkan Konus

**Affiliations:** 1 Department of Endodontics, Faculty of Dentistry, Firat University, Elazig 23119, Turkey; navci@firat.edu.tr; 2 Department of Software Engineering, Firat University, Elazig 23119, Turkey; okatar@firat.edu.tr; 3 Department of Endodontics, Faculty of Dentistry, Yozgat Bozok University, Yozgat 66100, Turkey; furkan.konus@bozok.edu.tr

**Keywords:** apical radiolucency, small-object detection, severity weighting, quadratic weighted kappa, test-time augmentation, dental informatics, computer-aided diagnosis

## Abstract

**Background/Objectives:** Apical periodontitis is among the most prevalent dental pathologies, and its radiographic severity is graded on panoramic radiographs using the ordinal periapical index (PAI 3 < 4 < 5). Existing computer-aided systems typically treat these grades as unrelated classes and down-sample the radiograph to a fixed input, which erases the small periradicular radiolucencies that define early disease. This study evaluated a lesion-preserving, ordinal-aware YOLO framework for the simultaneous detection and severity grading of periapical lesions. **Methods:** A total of 3924 expert-annotated panoramic radiographs (PAI 3/4/5) were processed with an adaptive lesion-preserving tiling algorithm that mapped each radiograph onto native-resolution 640 × 640 tiles so that every lesion appeared intact within exactly one tile, yielding 4641 tiles. Six consecutive YOLO generations (YOLOv8, YOLOv9, YOLOv10, YOLO11, YOLO12, and YOLO26) were benchmarked under one identical pipeline on a locked hold-out test set of 985 lesions, of which 628 were PAI 3, 274 PAI 4, and 83 PAI 5. YOLO12m, which attained the highest point estimates and the smallest run-to-run variation, was then progressively augmented with an ordinal-aware detection loss, a high-resolution P2 head, severity class-weighting and test-time augmentation. Localization was measured with COCO mAP and severity grading with severity-weighted average precision (Sev-wAP), quadratic weighted kappa (QWK), a catastrophic off-by-two error rate, and per-grade recall. **Results:** Lesion-preserving tiling improved detection for every generation, raising the mAP@0.50 of YOLO12m from 0.494 to 0.615, the highest point estimate among the six generations, although the margin over the next two was within the resolution of this test set. Cumulatively adding the ordinal loss, the P2 head, severity weighting, and test-time augmentation raised mAP@0.50 to 0.664, mAP@0.50:0.95 to 0.333, Sev-wAP to 0.704, and QWK to 0.780. PAI 5 average precision rose from 0.268 to 0.331 and PAI 5 recall from 0.602 to 0.663, 55 of 83 severe lesions, while catastrophic confusion between mild and severe lesions stood at 0.41%, 4 of the 985 reference lesions. **Conclusions:** Coupling lesion-preserving tiling with ordinal severity-aware detection improves localization and severity-weighted precision by resolvable margins, shifts every severe-grade measure in a clinically preferable direction, and provides a reproducible reference protocol for comparing detector architectures on this task.

## 1. Introduction

Inflammatory conditions of the periapical tissues are a central concern in clinical dentistry, bearing directly on endodontic treatment planning, tooth prognosis, and the long-term maintenance of oral health [1]. Among them, apical periodontitis is of particular clinical importance. It is a common disease of dentition, a principal reason for tooth extraction and an indication for root canal treatment [2]. Apical periodontitis is an inflammatory disorder marked by progressive destruction of the periradicular bone, arising in response to microbial infection of the root canal system [3]. Radiographically, it presents as a periapical radiolucency that enlarges as the surrounding alveolar bone is resorbed. To grade the severity of this appearance in a standardized and reproducible way, the periapical index (PAI) is widely used [4]. The PAI is a five-point ordinal scale on which higher scores denote a wider and more destructive lesion [5]. The range most relevant to routine panoramic screening spans PAI 3, PAI 4, and PAI 5, corresponding to changes in bone structure with some mineral loss, periodontitis with a well-defined radiolucent area, and severe periodontitis with exacerbating features. A defining property of the scale is that its grades are ordinal rather than nominal: they are arranged along a single axis of increasing severity [6]. Because apical periodontitis is frequently asymptomatic and affects a substantial proportion of adults, large numbers of periapical lesions must be detected and graded on radiographs obtained during routine dental care [7].

This clinical silence is precisely what makes the disease consequential: an undetected or incorrectly graded lesion can enlarge over months or years, undermine the support of adjacent teeth, and precipitate acute exacerbations or spreading odontogenic infection [8]. Because the grades of the PAI scale are ordinal, the clinical cost of misjudging severity is markedly asymmetric: reading a severe lesion as an early one may delay the endodontic or surgical intervention that the disease requires, whereas disagreement between two neighbouring grades less often alters the chosen course of management [9]. This asymmetry is a premise of the present work rather than a quantity it measures, and it is the kind of caution-weighted cost structure that risk-averse frameworks for medical artificial intelligence formalize [10]. Any automated grading system intended for clinical use should therefore respect the ordering of the scale rather than treat its grades as interchangeable labels. In everyday practice, detection and grading rest almost entirely on radiographic interpretation. Panoramic radiography is a widely used two-dimensional imaging modality for this purpose, delivering a low radiation dose, remaining cost-effective, and depicting both jaws in a single exposure [11]. Its diagnostic yield is limited, however, by the subjectivity of visual assessment, and pronounced interobserver variability has been reported [12]. This variability is greatest for early and subtle lesions, where anatomical superimposition, geometric distortion, and the collapse of a three-dimensional anatomy onto a two-dimensional plane reduce lesion conspicuity and blur the margins on which accurate grading depends [13]. Such subjectivity is a recognized constraint on diagnostic consistency and reproducibility, and this is the principal motivation for the artificial-intelligence-based computer-aided diagnostic systems now being developed to improve the accuracy, objectivity, and standardization of interpretation in dental radiology [14].

Deep-learning-based convolutional neural networks have proven highly effective for analysing dental radiographs, learning discriminative features automatically, and performing localization and classification directly from annotated images [15]. Among these methods, the “you only look once” (YOLO) family of object detectors [16] has drawn considerable attention. These localise and classify anatomical structures and pathologies on radiographic images with high accuracy while operating in real time. The architecture has also evolved rapidly across successive generations, progressing from the anchor-free design of YOLOv8 to the programmable gradient information of YOLOv9 and the non-maximum-suppression-free formulation of YOLOv10 to the increasingly attention-centric YOLO11, YOLO12 and YOLO26 versions. Each generation offers an object-detection approach that can serve as an objective decision-support tool, one that integrates into routine panoramic workflows without any change to standard imaging protocols.

A first line of research has applied deep learning to periapical assessment on three-dimensional cone-beam computed tomography (CBCT), where convolutional networks and commercial platforms have been used to detect periapical pathosis, segment lesions, and estimate their volume, with good agreement against expert annotation [17,18,19,20,21]. These studies demonstrate the strength of deep learning for periapical assessment, but they do so within a three-dimensional setting that is far less common in routine radiographic screening than two-dimensional imaging.

A second and closely related line of research has focused on two-dimensional panoramic and periapical radiographs, where convolutional detectors, segmentation networks, and commercial systems have been applied to the presence or absence of apical lesions, in several cases reaching agreement comparable to that of experienced examiners [22,23,24,25,26,27,28,29]. A smaller group has gone further and predicted the severity grade itself [30,31,32,33,34].

Building on these advances, several opportunities remain open. Most existing approaches have been developed and evaluated with a single detector or model under study-specific protocols. Although a comparison across successive YOLO generations has recently begun to appear for intraoral radiographs [33], these rapidly evolving generations have not yet been compared with one another under a common panoramic periapical benchmark. Lesion severity, moreover, has most often been represented as the presence or absence of a lesion or as a nominal category such that the ordinal nature of the PAI scale has remained relatively underexplored within the training objective itself. These reported figures should also be read with the design of the underlying collections in mind, since most are assembled around teeth or radiographs already known to carry a finding and few are validated outside the centre that produced them, a qualification that applies to the present study as much as to the work it builds on. A further practical consideration is that panoramic radiographs are commonly down-sampled to a fixed network input, a step that can reduce the visibility of the small periradicular radiolucencies associated with early disease. These observations motivated the present study, which developed and evaluated a lesion-preserving tiling and ordinal severity-aware framework built on the YOLO architecture for the automatic detection and severity grading of periapical lesions on panoramic radiographs.

The main contributions of this study can be summarized as follows.

- A lesion-preserving tiling algorithm is proposed that maps each panoramic radiograph onto native-resolution 640 × 640 tiles such that every annotated lesion appears intact within exactly one tile without any lesion being cropped, duplicated, or discarded, and this scheme consistently improves detection across all evaluated YOLO generations.
- A systematic benchmark of six consecutive YOLO generations (YOLOv8, YOLOv9, YOLOv10, YOLO11, YOLO12, and YOLO26) is conducted for periapical lesion detection under one identical pipeline, data split and evaluation protocol, providing a fair reference for architecture selection.
- An ordinal severity-aware framework is introduced, comprising a severity-weighted average precision and an ordinal metric suite together with an ordinal-aware detection loss, which penalizes distant misgrades more heavily than adjacent ones, together with a severity weighting that recovers the sensitivity to severe lesions the smoothing costs when it acts alone.
- To the best of our knowledge, this is the first study to combine lesion-preserving tiling with an ordinal severity-aware detection objective for the grading of periapical lesions on panoramic radiographs. Neither element is new on its own, and the contribution lies in that conjunction and in the evaluation protocol that accompanies it rather than in any single component.

## 2. Materials and Methods

This was a retrospective secondary analysis of a publicly available dataset of panoramic radiographs in which periapical lesions had been annotated at three grades of the PAI. It was a methodological study that reported model performance on a single locked hold-out partition and evaluated no clinical outcome. Each radiograph was first processed by an adaptive lesion-preserving tiling algorithm that decomposes the image into native-resolution tiles while keeping every lesion intact such that the small radiolucencies of early disease are retained at full detail. Six consecutive generations of the YOLO detector were then trained and evaluated on these tiles under one identical pipeline, allowing the architectures to be compared fairly and one of them to be selected as the backbone for the components that follow. This detector was subsequently extended with the components that form the core of this work: an ordinal-aware detection loss, a high-resolution P2 detection head, a severity-based weighting of the classification loss, and test-time augmentation. Model performance was assessed on a locked hold-out test set using both conventional localization metrics and a dedicated ordinal metric that reflects the clinical importance of correct severity grading.

**Figure 1:**
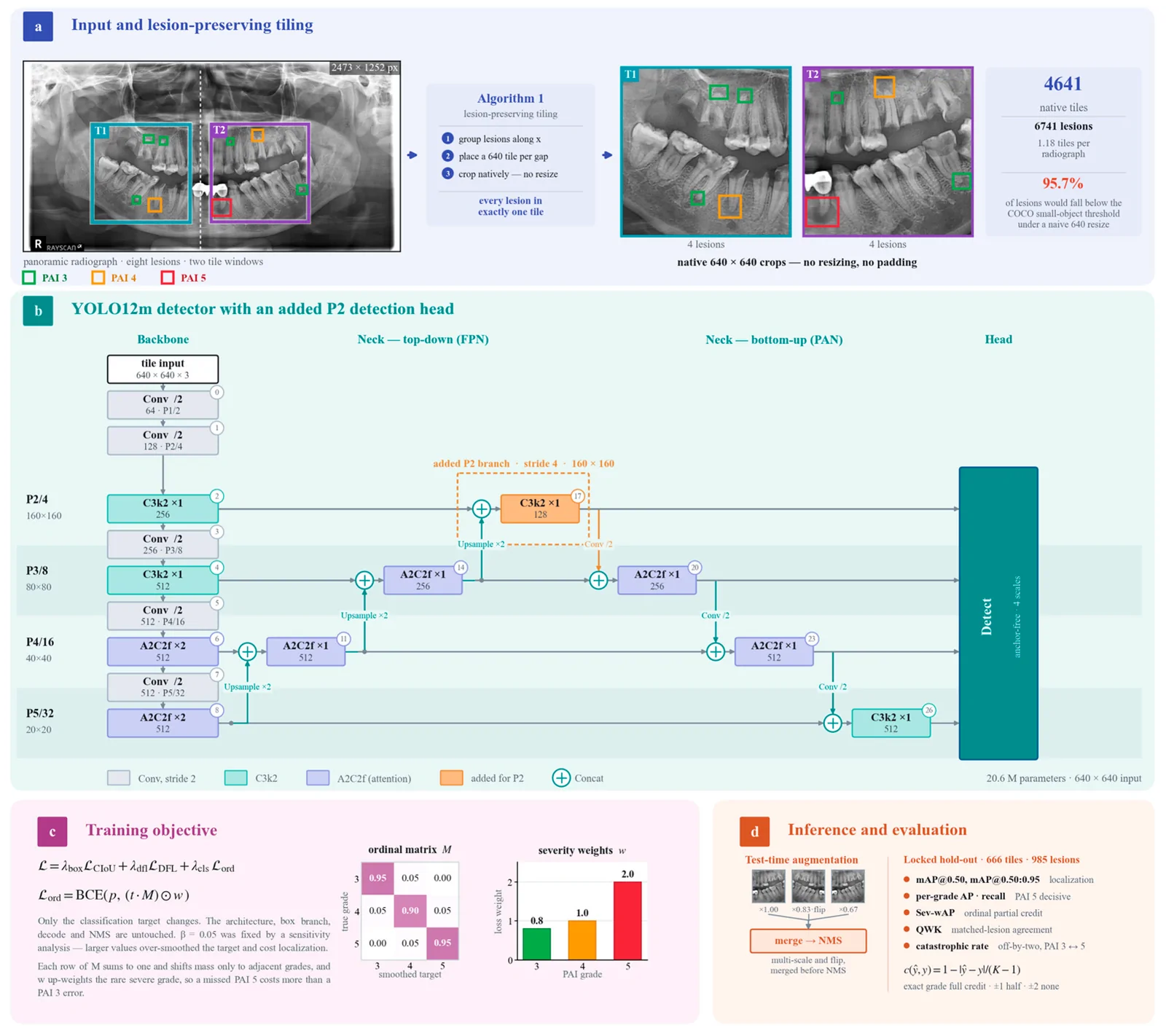
Overview of the proposed lesion-preserving tiling and ordinal-aware YOLO framework. (**a**) A panoramic radiograph is decomposed into native 640 × 640 tiles, cropped without resizing or padding and placed so that every lesion falls inside exactly one tile, which yields 4641 tiles carrying all 6741 lesions and retains detail that a naive resize would erase. (**b**) The YOLO12m detector extended with a P2 branch, adding a stride-4 level at 160 × 160 beside the default P3, P4 and P5 levels and bringing the model to 20.6 M parameters, with only the neck and the head extended. (**c**) The training objective, in which the ordinal matrix M shifts a small share of the target mass onto adjacent grades and the severity vector w raises the cost of an error on the rare severe grade, both acting on the classification term alone. (**d**) Inference and evaluation, where three test-time views are pooled by a single non-maximum suppression and the locked hold-out set of 666 tiles and 985 lesions is scored with conventional localization metrics beside the ordinal suite.

The overall workflow is presented in Figure 1, and each component is detailed in the following subsections.

### 2.1. Dataset

**Figure 2:**
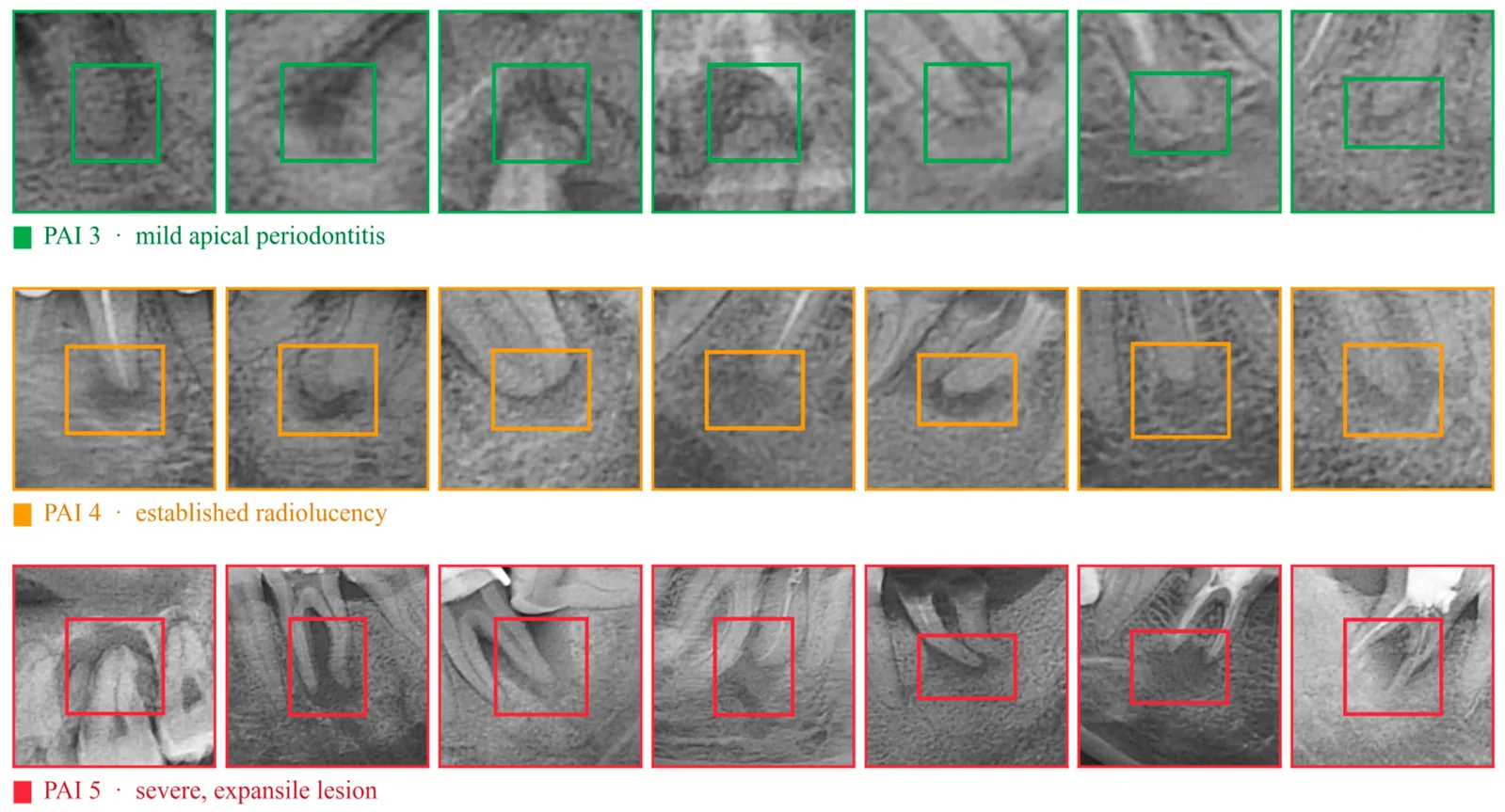
Representative periapical lesions for each grade of the PAI. Each row collects seven examples of a single grade, cropped from the native-resolution radiograph as a square centred on the annotated bounding box whose side is 2.1 times the larger side of that box, so that the surrounding anatomy is visible, and then scaled to a common panel size, with the box drawn in the colour of its grade. A typical lesion box has a median side of 60 pixels with an interquartile range of 50 to 77, so a typical crop covers about 126 pixels of the original radiograph and the panels are not all at the same magnification.

This study used a publicly available collection of panoramic radiographs with apical periodontitis lesions [35]. The radiographs were acquired at a dental centre affiliated with Hanoi Medical University between January 2016 and March 2021. They were captured on a single Sirona Orthophos XG (Dentsply Sirona, Bensheim, Germany) unit and stored as JPEG images at 235 dpi, and were selected from a larger collection of 16,519 radiographs gathered at the same centre on the basis that each carried at least one periapical lesion, the data descriptor stating no further inclusion criterion. All radiographs therefore originate from one institution, one device and one acquisition protocol, which bounds the population and equipment generalizability of every result reported here. The collection is also distributed with scaled, mirrored, rotated and noise-augmented duplicates of each radiograph. These were discarded and only the original images were used, since retaining copies of the same radiograph across different partitions would have produced leakage. The collection comprises 3924 panoramic radiographs, each containing at least one periapical lesion, with 6741 lesions annotated in total. All counts reported here were obtained directly from the released annotation files. Every lesion was marked with an axis-aligned box and a PAI grade under the consensus protocol described below. Only the three pathological grades appear in the data, since PAI 1 and PAI 2 denote normal periapical structures and were treated as healthy at source. Representative examples of the PAI 3, PAI 4 and PAI 5 grades are shown in Figure 2.

The annotations followed a strict consensus rule. Three dentists, each with more than five years of experience, annotated independently after calibrating on fifty radiographs. A lesion was retained only when boxes from at least two of the three readers overlapped above an intersection over union (IoU) of 0.8, and in that case the annotation of the agreeing majority was adopted as the reference. Radiographs on which all three readers disagreed were discarded at source, as were lesions that failed the overlap criterion. The reference standard is therefore deliberately conservative and describes radiographically unambiguous disease by construction.

Two consequences follow. The first is a spectrum bias: because equivocal appearances were removed before the collection was released, the performance reported below is an estimate on consensus-verified lesions and is likely optimistic relative to unselected clinical practice, where borderline lesions are precisely the cases for which assistance is most wanted. The second concerns inter-reader agreement. The released data contain a single consensus annotation per lesion. The individual readers’ annotations are not distributed and the data descriptor reports no agreement coefficient, so the annotators’ pairwise agreement cannot be recovered from this material. Section 2.11 describes a reader study conducted to supply the human comparator this omission would otherwise leave missing.

Lesion geometry motivates the emphasis on input representation. The radiographs are stored at several native resolutions, so all preprocessing was defined relative to each image’s own dimensions rather than a fixed pixel grid. A typical lesion box has a median side of 60 pixels, with an interquartile range of 50 to 77, and occupies a median of 0.114% of the radiograph. When a full radiograph is resized to the 640 × 640 input common to these detectors, the median lesion spans only about 16 pixels, and at that scale 95.7% of all lesions fall below the 32 × 32 threshold of the COCO small-object definition [36]. The lesion-preserving tiling of Section 2.2 is designed to prevent this loss of detail.

**Figure 3:**
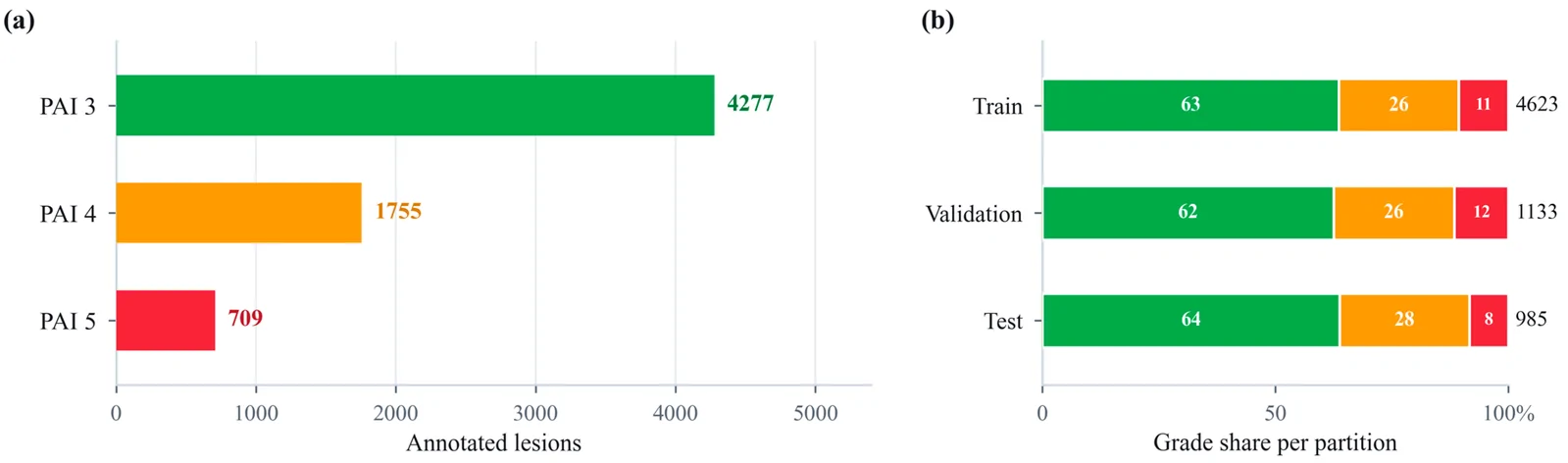
Distribution of the annotated lesions across the three PAI grades. (**a**) Number of lesions per grade, showing the long-tailed structure in which PAI 3 dominates and PAI 5 is scarce. (**b**) Grade composition of the training, validation and test partitions, with the number of lesions in each partition given at the right. The three partitions follow a similar grade composition, with the severe grade slightly less frequent in the test partition.

The grade distribution is long-tailed, as summarized in Table 1 and shown in Figure 3. PAI 3 accounts for 63.45% of lesions and PAI 5 for only 10.52%, a head-to-tail ratio of about six to one, so the grade that matters most in screening is the scarcest in the data. The partitions follow a similar composition: the locked test set contains 985 lesions, of which 628 are PAI 3, 274 are PAI 4 and 83 are PAI 5. That last figure bears directly on how the severe-grade results below should be read, since every PAI 5 rate reported in this study rests on those 83 lesions. This imbalance motivates both the severity weighting in Section 2.6 and the per-grade reporting used throughout.

### 2.2. Lesion-Preserving Tiling

The radiographs are about 2444 × 1292 pixels, whereas the detectors take a 640 × 640 input. A full-image resize therefore contracts each axis by roughly 3.8. A 40-pixel radiolucency survives as barely 10 pixels, below the scale at which a stride-8 head responds reliably. Sliding-window cropping avoids the contraction, but it cuts some lesions at window boundaries and duplicates others across overlaps. This corrupts both the training signal and the evaluation count.

The tiling is governed by a single hard constraint: every lesion must appear intact in exactly one emitted tile. Writing L for the set of lesions in a radiograph, $b_{l}$ for the box of lesion l and $W_{k}$ for the k-th emitted window, this requirement is formalized in Equation (1).

(1) $$\forall l\in L,\;\exists !\;k\;\in \;\left\{1,\;\ldots ,\;n\right\}:\;b_{l}\;\subseteq \;W_{k}$$

Existence forbids discarding a lesion, uniqueness forbids duplicating it and the subset relation forbids cutting it. The three failure modes of naive cropping are thus excluded by construction.

The proposed algorithm proceeds in three steps. Lesions are first grouped greedily along the horizontal axis, the dominant extent of a panoramic image and the axis along which related lesions cluster within a quadrant, and a group is admitted only while its combined horizontal and vertical extents stay within the tile side such that every window is a native-resolution crop taken with no resizing and no padding. The largest single lesion in this collection measures 467 pixels on its longer side, so even a group of one fits inside the tile and no emitted window is ever rescaled. The window is then placed so that its edges fall in the gaps that separate the group from its neighbours rather than on a fixed grid, which is what allows each lesion to be kept whole, and its origin is clamped so that the window never leaves the radiograph. Third, lesion-free regions emit no tile, so background does not dilute training, a design choice whose cost at inference time is examined in Section 3.7. The placement rule, the boundary case in which no admissible gap exists, and the fallback that would apply to a group wider than the tile are stated line by line in Algorithm 1.
**Algorithm 1.** Adaptive lesion-preserving tiling**Input:** radiograph I of size W × H; lesion boxes B = {bℓ = (xℓ, yℓ, wℓ, hℓ, classℓ)} with ℓ ∈ L; tile side T = 640. **Output:** a set of tiles, each a T × T image, in which every lesion is contained in exactly one tile. 1: **sort** B in ascending order of the left coordinate x_ℓ_ 2: G ← ∅ 3: **for** each b_ℓ_ ∈ B **do**  4: find the first group G_k_ ∈ G whose bounding box, after adding b_ℓ_, still fits within T × T  5: **if** such a group exists **then** G_k_ ← G_k_ ∪ {b_ℓ_}  6: **else** append the singleton {b_ℓ_} to G 7: **end for** 8: **for** each group G_k_ ∈ G **do**  9: compute w_k_, h_k_ and set s_k_ ← max(T, w_k_, h_k_)  10: a_k_ ← min{ x_ℓ_: ℓ ∈ G_k_ }; e_k_ ← max{ x_ℓ_ + w_ℓ_: ℓ ∈ G_k_ }  11: λ ← max({ x_j_ + w_j_: b_j_ entirely left of G_k_ } ∪ { 0 })  12: ρ ← min({ x_j_: b_j_ entirely right of G_k_ } ∪ { W })  13: lo ← max(e_k_ − s_k_, λ, 0)  14: hi ← min(a_k_, ρ − s_k_, W − s_k_)  15: **if** lo ≤ hi **then** x_k_ ← ⌊(lo + hi)/2⌋  16: **else** x_k_ ← clip(c_k_ − s_k_/2, 0, W − s_k_)  17: y_k_ ← clip(d_k_ − s_k_/2, 0, H − s_k_)  18: P ← I[ y_k_: y_k_ + s_k_, x_k_: x_k_ + s_k_ ]  19: **if** s_k_ > T **then** P ← resize(P, T × T)  20: **for** each b_ℓ_ ∈ G_k_ **do**    21: emit tile P with box (x_ℓ_ − x_k_, y_ℓ_ − y_k_, w_ℓ_, h_ℓ_) and label class_ℓ_  22: **end for** 23: **end for**

**Figure 4:**
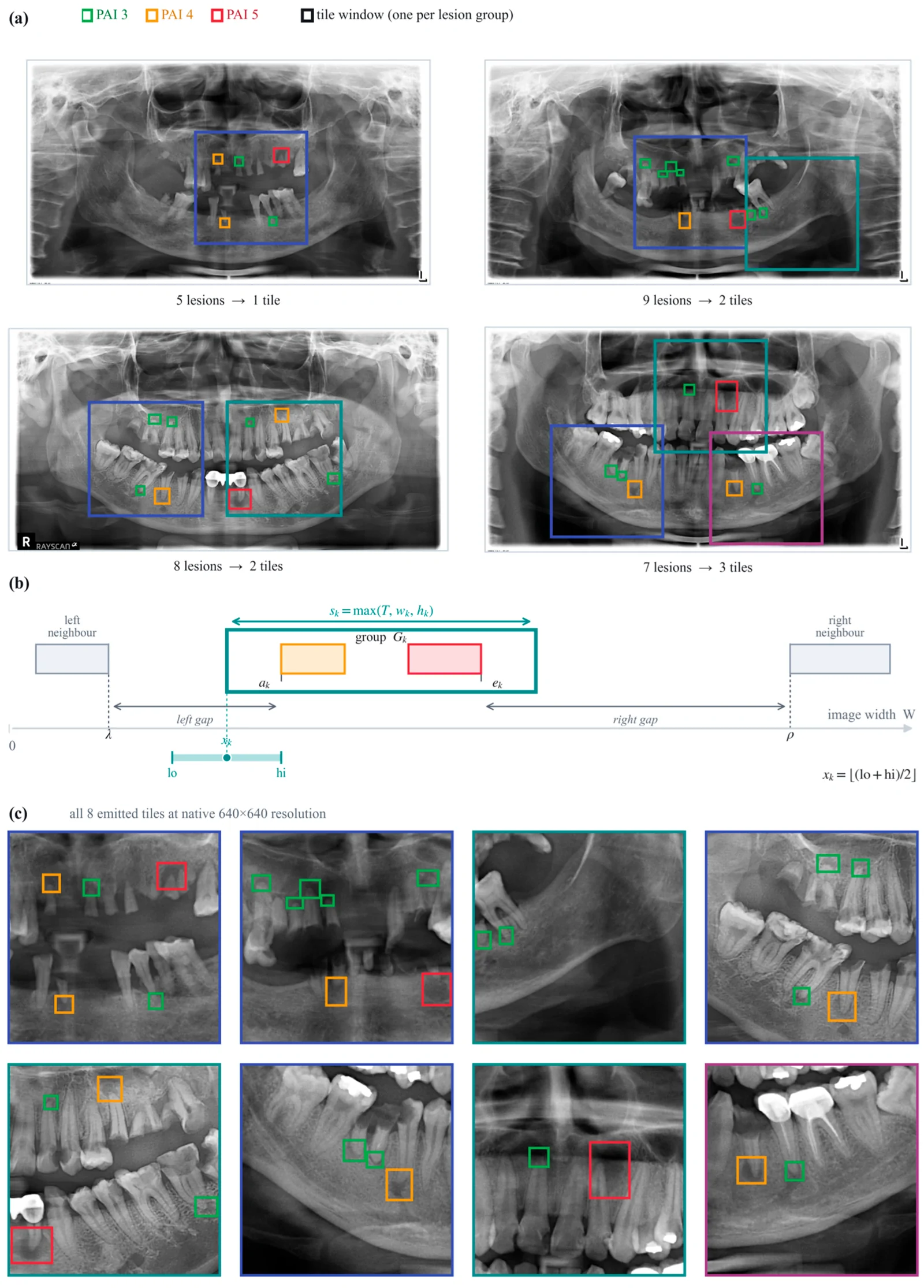
Tile generation on panoramic radiographs. (**a**) Four radiographs from the dataset, with every annotated lesion drawn in the colour of its grade and the emitted tile windows outlined, one window per lesion group. The number of lesions and the number of tiles produced are given beneath each radiograph. (**b**) Placement of the horizontal origin. The window side covers the group, and the origin is taken at the midpoint of the admissible interval bounded by lo and hi, which keeps the tile edges inside the gaps that separate the group from its left and right neighbours. (**c**) All eight tiles emitted from these four radiographs, each at the native 640 × 640 resolution the detector receives.

Applied to the 3924 radiographs, the procedure produced 4641 tiles carrying all 6741 lesions, an average of 1.18 tiles per radiograph and 1.45 lesions per tile. The mapping is exhaustive and non-redundant, so predictions compose back to the radiograph without double counting and the totals in Table 1 hold exactly. Figure 4 shows the scheme on four representative radiographs, at the scale the detector actually sees.

### 2.3. Detection Models and Benchmark

Six consecutive generations of the YOLO family were benchmarked at the medium (m) scale, spanning the anchor-free C2f baseline of YOLOv8 [37] to the 2026 release YOLO26 [38], with YOLOv9 [39], YOLOv10 [40], YOLO11 [41] and YOLO12 [42] between them. The six generations and their principal architectural characteristics are given in Table 2. They trace the main design shifts of the family: the programmable gradient information and GELAN backbone of YOLOv9, the NMS-free end-to-end training of YOLOv10, the C3k2 blocks of YOLO11 and the attention-centric backbone of YOLO12. All six were trained under one identical Ultralytics pipeline, with the same optimizer, schedule, augmentation policy and COCO-pretrained initialization. The comparison therefore isolates the effect of architectural evolution rather than training heuristics. Every model exported its predictions in a common COCO format, scored by a single evaluation harness, so that every generation was measured identically.

### 2.4. Ordinal-Aware Detection Loss

A standard detection head treats the three severity grades as independent classes. Its classification branch minimizes a per-class binary cross-entropy against a one-hot target, so every misgrade carries the same cost. This conflicts with the meaning of the PAI scale, whose grades are ordered. A two-step error, in which a PAI 3 lesion is read as PAI 5, is clinically far more serious than a one-step error between adjacent grades. The ordinal-aware loss writes this ordering directly into the training target and leaves the network architecture unchanged.

The mechanism is a soft relabelling of the classification target. A one-hot grade label cedes a small factor β of its mass to each immediate neighbour and keeps zero on distant grades. Writing K for the number of grades, $N\left(i\right)$ for the ordinal neighbours of grade i and $M_{ij}$ for the target mass that a lesion of grade i places on grade j, the smoothing matrix M is defined in Equation (2).

(2) $$M_{ij}=\left\{\begin{array}{ll} 1-\beta \left|N\left(i\right)\right|, & \;\mathrm{if}\;i=j\\ \beta , & \;\mathrm{if}\;\left|i-j\right|=1\\ 0, & \;\mathrm{otherwise} \end{array}\right.$$

Each row of M sums to one by construction, so the relabelling redistributes the target mass without changing its total. For a lesion of grade g with one-hot vector t, the soft target is the g-th row of M, that is $\hat{t}=t\;M$. With K=3 and β=0.05, the matrix takes the concrete form given in Equation (3).

(3) $$M=\left[\begin{array}{ccc} 0.95 & 0.05 & 0\\ 0.05 & 0.90 & 0.05\\ 0 & 0.05 & 0.95 \end{array}\right]$$

The soft target replaces the one-hot target in the classification loss, which is otherwise the same binary cross-entropy used by the detector. Writing ${CE}_{c}$ for the cross-entropy of grade c and $p_{c}$ for the probability the detector assigns to that grade, the loss is given in Equation (4).

(4) $$L_{\mathrm{ord}}=\sum_{c=1}^{K}{\mathrm{CE}}_{c},\;\;{\mathrm{CE}}_{c}=-\left[{\hat{t}}_{c}\;\log\;p_{c}+\left(1-{\hat{t}}_{c}\right)\;\log\;\left(1-p_{c}\right)\right]$$

The effect is asymmetric across error types. An adjacent grade receives a small but positive share of the target mass, so a one-step confusion is given partial credit and its loss is softened. A distant grade receives no mass, so a two-step confusion is penalized in full. The gradient therefore pushes hardest against the clinically critical PAI 3 to PAI 5 error, while the bulk of the signal still concentrates on the true grade. Only the classification target is modified. The smoothing factor was deliberately small, at β=0.05, so that most of the target mass stays on the true grade and the ordinal signal refines the severity decision rather than dominating it. A value of 0.10 was also trained under the identical protocol and was worse on every axis, lowering mAP@0.50 from 0.609 to 0.580, Sev-wAP from 0.691 to 0.656 and raising the catastrophic rate from 0.41% to 1.02%, so the smaller setting was retained. The full sensitivity analysis for both hyperparameters is reported in Section 3.3. This loss governs how errors between grades are penalized. The separate problem of how often each grade is seen during training is addressed by the severity weighting of Section 2.6.

### 2.5. High-Resolution P2 Detection Head

A panoramic lesion is small relative to the images the detectors were designed for. Even after lesion-preserving tiling keeps each lesion at native resolution, an early PAI 3 lesion often occupies only a small fraction of a 640 × 640 tile, and the mild grade contains the smallest lesions in the collection, with a median box side of 53 pixels against 75 for PAI 4 and 117 for PAI 5, so it is where a coarse prediction grid is most likely to fail. A detector that predicts only at coarse strides can miss such lesions, because a small object may fall between the cells of a coarse grid or be described by too few features to separate it from the trabecular background.

The default head of the YOLO detectors predicts at three pyramid levels, P3, P4 and P5, with strides of 8, 16 and 32. For a 640 input, these correspond to grids of 80 × 80, 40 × 40 and 20 × 20. The smallest stride, 8, sets the finest resolution at which an object can be localized. To sharpen the response to small lesions, a fourth level, P2, was added at stride 4, one step finer than the default. The grid resolution at each level follows the input side S and the level stride $s_{l}$, as given in Equation (5).

(5) $$N_{l}=\frac{S}{s_{l}}$$

**Figure 5:**
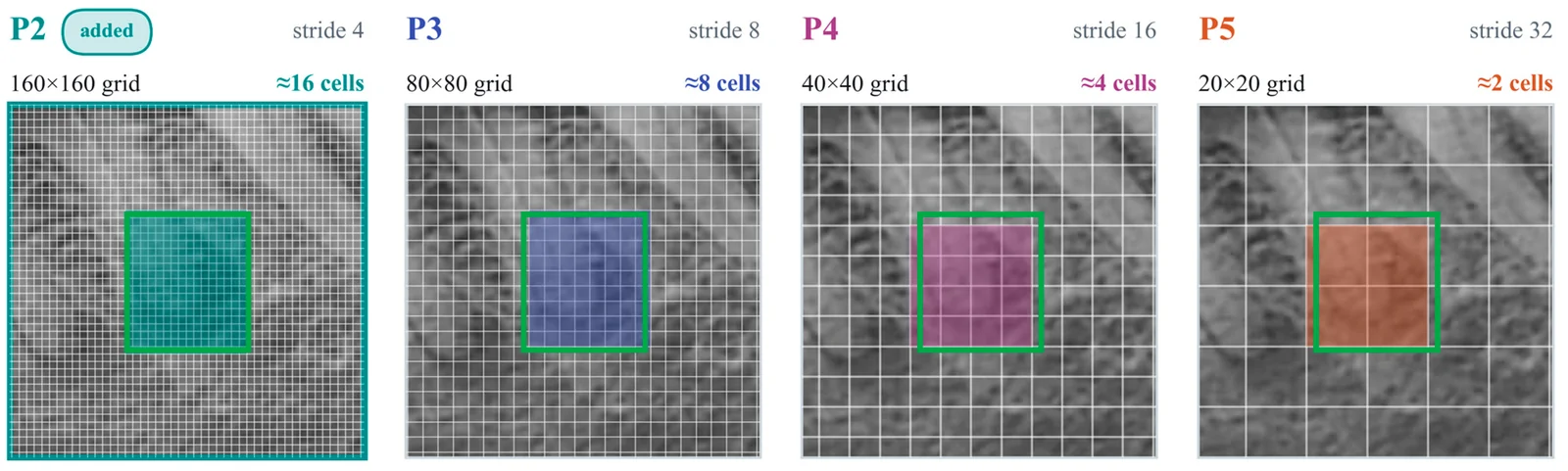
The four detection levels of the P2-augmented model, shown on the same PAI 3 lesion (green box). Each panel overlays the prediction grid of one pyramid level, with the cells covering the lesion tinted in the level colour. The added P2 level, at stride 4, resolves the lesion into about sixteen cells per axis, whereas the coarsest default level P5, at stride 32, covers it with only about two, so a small lesion can fall between the cells of the coarse grids that the finer P2 head recovers.

With S = 640, the added P2 level yields a 160 × 160 grid, twice the per-axis resolution and four times the cell count of the previously finest level P3. A lesion is therefore covered by more predicting cells and its centre is quantized more finely, which improves both recall and localization for the smallest grades. The four levels are shown in Figure 5.

The extra level was built from C3k2 blocks, chosen to keep the higher-resolution feature map within the memory budget of the training batch. The resulting model has 20.6 M parameters, so the finer head adds capacity without changing the scale class of the detector. Only the neck and the head are extended, and the backbone and the ordinal training objective of Section 2.4 are left unchanged.

### 2.6. Severity Class-Weighting

The three grades are far from balanced. As Table 1 shows, PAI 3 accounts for roughly two-thirds of the lesions while PAI 5 accounts for about one-tenth, a head-to-tail ratio of about six to one. Under a plain classification loss, the abundant mild grade dominates the gradient and the rare severe grade contributes little to training. This inverts the clinical priority, since the severe grade is the one whose detection carries the greatest consequence. The practical result is a systematic loss of recall on PAI 5.

This is a problem of frequency rather than of ordering and it is therefore addressed separately from the ordinal-aware loss of Section 2.4. To counter it, each grade is given a fixed weight $w_{c}$ that scales its contribution to the classification loss, so that an error on the rare severe grade counts for more than an error on the abundant mild grade. Applying these weights to the per-grade term ${CE}_{c}$ of Equation (4) yields the severity-weighted loss of Equation (6).

(6) $$L_{\mathrm{sev}}=\sum_{c=1}^{K}w_{c}\;{\mathrm{CE}}_{c}$$

The weights were set to w=(0.8, 1.0, 2.0) for grades PAI 3, PAI 4 and PAI 5. The severe grade is doubled relative to the middle grade and the mild grade is slightly reduced, which raises the training emphasis on PAI 5 without silencing the other grades. The setting is deliberately soft. An aggressive inverse-frequency scheme, with weights of about (0.53, 1.28, 3.17), was also tried, but it lowered overall detection accuracy without improving the severe grade, so the gentler weighting was retained. Like the ordinal smoothing of Section 2.4, the weighting acts only on the classification term and leaves the box regression and the rest of the detection pipeline unchanged. The weighted classification term enters the composite objective alongside the unchanged box-regression and distribution-focal terms, with the Ultralytics default balance retained so that the ablation isolates the proposed components rather than a retuned loss, as given in Equation (7).

(7) $$L=\lambda_{\mathrm{box}}L_{\mathrm{box}}+\lambda_{\mathrm{cls}}L_{\mathrm{cls}}+\lambda_{\mathrm{dfl}}L_{\mathrm{dfl}}$$

### 2.7. Test-Time Augmentation

Test-time augmentation is an inference-only step that leaves the trained weights untouched. Each tile is passed through the detector several times under simple label-preserving transforms and the resulting detections are merged. The transforms combine a horizontal flip with a small set of scale changes. A horizontal flip is anatomically valid for a panoramic view whose left and right sides are broadly symmetric, so it introduces no implausible geometry, and multi-scale evaluation lets a lesion be seen at more than one effective size.

**Figure 6:**
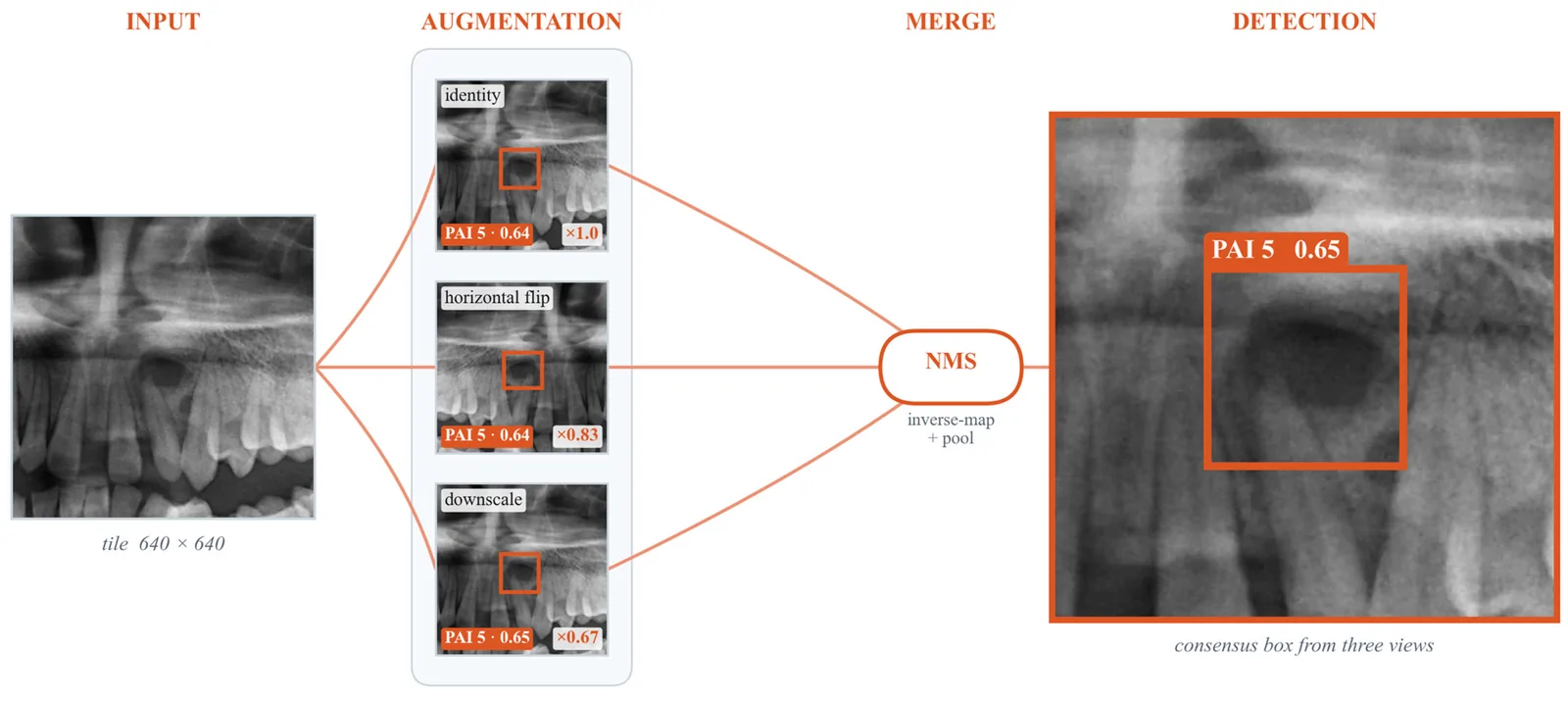
The test-time-augmentation pipeline on a real test tile with a severe (PAI 5) lesion. The input tile is augmented into the three views the detector uses, the identity, a horizontal flip at 0.83 scale and a downscale to 0.67, and the network is run on each, with its box, grade and confidence shown. A panoramic view is broadly left-right symmetric, so the flip is anatomically valid. The three detections are mapped back to tile coordinates and pooled by a single non-maximum suppression into the consensus detection on the right, enlarged on the lesion and graded PAI 5. Every box is a real detector output.

The final model uses the three views of the detector’s built-in augmentation, the tile at full scale, a horizontally flipped tile at 0.83 scale, and the tile reduced to 0.67 scale. Each view is passed through the network, its predictions are mapped back to the original tile coordinates by the inverse transform and the pooled predictions are reduced by a single non-maximum suppression. Figure 6 traces the three views on a severe lesion, where all three agree and the suppression returns one confident PAI 5 detection.

Pooling several views shifts the whole operating curve towards sensitivity: at a fixed confidence of 0.30, it raises true positives from 745 to 812 while also raising false positives from 255 to 497, and because average precision integrates over confidence, the net effect is an improvement at every grade, with the largest gain on the severe lesions a single-view detector is most likely to miss. It is a recall-favouring rather than a suppressing operation. Test-time augmentation is the only inference-time component. Every training-time comparison in Section 3 is free of it, and it is reported both on the final model and as a reference row in the cumulative ablation of Section 3.3, on the tiled baseline, so that its contribution can be read apart from any interaction with the components that precede it. To keep the higher memory cost of multi-view inference within the GPU budget, the augmented passes were run at a batch size of one, with the cache cleared between tiles.

### 2.8. Evaluation Metrics

The endpoints of this study are ordered rather than treated as interchangeable. Localization accuracy at mAP@0.50 is the primary endpoint and was fixed before the experiments, and it is the criterion on which the retained checkpoint of every run was chosen, computed on the validation partition alone. The ordinal measures defined below are secondary and descriptive, since they characterize how a model distributes its errors across the severity scale rather than how much disease it finds.

Localization was measured with the COCO average precision family, mAP@0.50, the primary mAP over IoU 0.50 to 0.95 and per-grade AP. These are nominal and score a severe lesion graded as mild exactly as one graded as its neighbour, so we add an ordinal suite. Its basis is a partial-credit rule: a detection d in the matched set M, localized at IoU at least 0.50 with predicted grade ŷ and reference grade y, earns a credit that decays linearly with the ordinal distance between the two and forfeits the remainder to the false-positive mass, as given in Equation (8).

(8) $$\begin{array}{l} c_{d}=1-\frac{\left|{ŷ}_{d}-y_{d}\right|}{K-1},\;d\;\in \;M\\ \mathrm{TP}=\sum_{d\;\in \;M}c_{d},\;\mathrm{FP}=\sum_{d\;\in \;M}\left(1-c_{d}\right)+\left|U\right| \end{array}$$

An exact grade earns full credit, a neighbour one half and the opposite extreme none while adding a full unit of false positive. Detections that match no reference form the unmatched set U and are counted as false positives, whereas a reference that no detection matches is a miss that lowers recall rather than raising the false-positive mass. Ranking detections by confidence and accumulating these quantities traces an ordinal precision–recall curve whose 101-point interpolated area is the severity-weighted average precision given in Equation (9).

(9) $$\begin{array}{l} r=\frac{\mathrm{TP}}{n_{\mathrm{ref}}},\;p=\frac{\mathrm{TP}}{\mathrm{TP}+\mathrm{FP}}\\ \mathrm{Sev}\text{-}\mathrm{wAP}=\frac{1}{101}\sum_{t\;\in \;R}p_{\mathrm{int}}\left(t\right),\;p_{\mathrm{int}}\left(t\right)=\max_{r\;\geq \;t}\;p \end{array}$$

Sev-wAP accumulates over all detections pooled rather than averaging across grades, so it reduces to the class-agnostic AP@0.50 of the same detector when every matched detection is graded correctly, which is 0.776 for the tiled baseline, and degrades with ordinal distance otherwise. It is therefore on a different scale from the class-averaged mAP@0.50 and the two should be read separately rather than against one another. Grade agreement over matched lesions is summarized by the quadratic weighted kappa, from the observed confusion matrix O and the matrix E expected under independence, as given in Equation (10).

(10) $$\kappa =1-\frac{\sum_{i,\;j}v_{ij}O_{ij}}{\sum_{i,\;j}v_{ij}E_{ij}},\;v_{ij}=\frac{{\left(i-j\right)}^{2}}{{\left(K-1\right)}^{2}}$$

The catastrophic error rate counts confusions spanning the full scale, a mild lesion called severe or the reverse, normalized over all reference lesions so it cannot be lowered by detecting less, as given in Equation (11).

(11) $$\mathrm{Catas}=\frac{\left|\left\{d\;\in \;M:\;\left|{ŷ}_{d}-y_{d}\right|=K-1\right\}\right|}{n_{\mathrm{ref}}}$$

The severity mean absolute error completes the suite. Over the matched set, it is the mean of the absolute ordinal distance between the predicted and the reference grade, so it reports the average size of a grading error on the scale itself rather than the frequency of any particular error type. Sev-wAP is an internally defined partial-credit construction: it is not comparable with the mean average precision reported in other studies, which counts a detection correct on localization alone, and it should be used only to compare configurations within this study. Nor has it been validated against any clinical outcome. It encodes an assumed cost structure, and the asymmetry that structure expresses is a design premise of this work rather than a quantity we measure.

The ordinal scoring these metrics share is driven entirely by the distance between the predicted and the reference grade. The partial credit awarded to a matched detection falls linearly with that distance, so an adjacent-grade prediction is worth half of a correct one and a two-grade prediction nothing, while the kappa penalty grows with its square, so a two-grade error costs four times an adjacent-grade one. Per-grade recall is reported throughout as the number of reference lesions of a grade that are both detected and assigned that grade, divided by all reference lesions of that grade, so that a lesion the detector never finds counts against recall rather than being excluded from its denominator. Emphasis falls on PAI 5, since an unreported severe lesion is the costliest error.

Localization at mAP@0.50, the severity-aware measures, and the per-grade rates reported in Section 3 carry interval estimates, and every comparison on which a claim of this paper rests is reported with an interval on the paired difference. The tables give point estimates so that configurations can be read against one another at a glance, and the corresponding intervals appear in the text wherever a difference is claimed. Because lesions arising from the same radiograph are not independent observations, and because the released data provide no patient identifiers with which a stronger grouping could be formed, intervals are obtained by a cluster bootstrap that resamples whole radiographs with replacement, 2000 times, so that all lesions belonging to one radiograph move together. Comparisons between configurations use the same resampling applied to both configurations simultaneously, and the interval is formed on the paired difference rather than on each configuration separately. A difference is described as resolvable only when its 95% interval excludes zero. Exact counts are given beside every rate, since several of the rates in this study rest on small numbers of lesions and a percentage alone would overstate the precision available.

Two further quantities describe the operating behaviour of the detector rather than its accuracy at a single threshold. The free-response operating characteristic traces localization sensitivity against the number of false positives per radiograph as the confidence threshold is swept, which is the form in which a screening operating point is normally chosen. The expected calibration error compares the confidence of a matched detection with the probability that its grade is correct, over ten equal-width confidence bins, and therefore measures whether the score can be read as a probability of correct grading rather than as a ranking.

### 2.9. Implementation Details

All experiments ran on a single Nvidia RTX 4090 GPU (NVIDIA Corporation, Santa Clara, CA, USA) with 24 GB of memory under the Ultralytics framework (version 8.4.90) with PyTorch (version 2.5.1) and CUDA (version 12.1). Models were trained on the lesion-preserving tiles at 640 × 640 with a batch size of 8 for up to 500 epochs from COCO-pretrained weights. Training stopped on a validation-loss criterion with a patience of 10 epochs, in practice between 28 and 62 epochs, and the checkpoint with the highest validation mAP@0.50 was retained. Tiles were partitioned at the radiograph level into 3195 training, 780 validation and 666 test tiles carrying 4623, 1133 and 985 lesions and drawn from 2690, 673 and 561 radiographs respectively, so that no two tiles from one radiograph straddle a split. The assignment was checked afterwards and the three partitions share no radiograph. The test partition was locked before training and used for neither early stopping nor checkpoint selection, and every performance result in Section 3 is computed on it, apart from the convergence curves of Section 3.4 and the diagnostic measurements that Section 3.7 identifies as made on validation. One qualification belongs with that statement. The two hyperparameters introduced in this work, the ordinal smoothing coefficient and the severity weights, were compared on the hold-out partition rather than on the validation partition, and Section 3.3 reports that comparison in full, as Section 3.2 does for the run-to-run comparison used to select the backbone. Their adopted values are therefore not independent of the test set, and the intervals of Section 2.8 carry no correction for that selection. We report both sweeps completely rather than only the retained settings, so that the size of every alternative is visible. Predictions were exported to COCO format and scored by the single harness of Section 2.8, so all six generations and all ablations are measured identically. Table 3 collects the full training configuration, shared unchanged by every model and ablation.

Augmentation is applied only within the training data loader and never to the validation or test partitions. Model selection, comprising early stopping and checkpoint selection, used the validation partition alone. The tiling itself was computed over the whole collection before the partitions were drawn, which requires a word of justification: it cannot transfer information between partitions, because the procedure operates on one radiograph at a time, uses only the lesion coordinates of that same radiograph, and assigns all tiles from a radiograph to the same partition. It is a per-image transformation applied uniformly rather than a preprocessing step fitted to data, and no statistic estimated from the training set enters it. The same discipline governs the inference pipeline of Section 2.10, whose window stride, merge rule and threshold were selected on the validation partition and applied once to the test partition.

No prospective sample size calculation was performed. The study was a retrospective secondary analysis that used all available data from a fixed public collection, so the sample size was determined by the dataset rather than chosen, and the relevant question is not what power was planned, but what the available material can resolve. The cluster bootstrap of Section 2.8 answers that directly: with 985 test lesions, mAP@0.50 is resolved to approximately plus or minus 0.04, whereas PAI 5 recall, which rests on 83 lesions, is resolved only to approximately plus or minus 0.10. Differences smaller than these are not detectable here, and this bound is stated wherever such a difference is discussed. On the training side, the same collection supplies 4623 lesions across 3195 tiles, of which 494 carry the severe grade, and the loss curves of Section 3.4 show every configuration reaching its best validation score and then flattening rather than diverging, so the training material is sufficient for the capacity used here. It is least sufficient for PAI 5, which contributes the fewest examples, and a larger collection could plausibly move that grade further than the components introduced here do.

### 2.10. Label-Free Inference Pipeline

The tiling of Section 2.2 takes lesion coordinates as its input. That is appropriate for building a training representation, but it means that a model evaluated on those tiles is scored on windows already centred on the lesions it is asked to find. To assess the framework as a deployable system rather than as a training representation, we implemented a second pipeline that uses no annotation at any point.

Each radiograph is covered by a dense grid of native-resolution 640 × 640 windows whose positions are determined by the image dimensions alone, with a stride of 320 pixels so that every location falls inside the interior of at least one window, and with the final window on each axis flush against the image edge so that no region is left unscanned. This yields on average 26.7 windows per radiograph against the 1.18 lesion-bearing tiles the annotation-driven scheme emits, so the detector is exposed to the whole radiograph, including all lesion-free anatomy. Detections from every window are mapped back to radiograph coordinates and merged across the radiograph by a single class-wise non-maximum suppression, which removes the duplicates that window overlap necessarily produces. Stride, merge rule, edge handling and the suppression threshold were chosen on the validation partition and applied once to the test partition. Predictions are scored against the radiograph-level reference standard, so the unit of evaluation is the radiograph rather than the tile. The cost of the scan was measured on the test partition: 234 s for all 561 radiographs, which is 0.42 s per radiograph on a single RTX 4090 that was simultaneously running another job, so the figure is an upper bound rather than a benchmark.

Evaluating this pipeline exposed a property of the tiling scheme that the annotation-driven evaluation could not reveal. Because lesion-free regions emit no tile, a detector trained on these tiles never encounters a pure-background window, whereas in a dense scan 65 to 73 per cent of windows contain no lesion at all. To test whether this mattered, the identical configuration was retrained with lesion-free tiles added, sampled from the same dense grid used at inference and drawn only from radiographs already assigned to the training partition, so that the split remains intact. Results for both variants are reported in Section 3.7.

### 2.11. Reader Study Design

The quadratic weighted kappa of a model is interpretable only against the agreement achievable by human readers on the same material, and the premise of this work is that PAI grading is observer-dependent. Since that agreement cannot be recovered from the released annotations, as explained in Section 2.1, we measured it directly.

A stratified sample of 249 lesions was drawn from the hold-out test set, comprising all 83 PAI 5 lesions together with 83 randomly selected lesions of each of the two remaining grades, so that the scarcest and most consequential grade is represented in full. Each lesion was presented as a blinded native-resolution crop centred on the annotation, with the lesion location marked by a single box of uniform colour that carried no information about grade, in an order randomised independently of grade. The three readers were the endodontist co-authors of this study, F.O., N.A., and F.K., and we state this because they are therefore not independent of the work. Each graded every lesion without access to the reference grade, to one another’s readings or to the model output, and without the opportunity to revise a decision once entered. None had been calibrated on the annotation convention of the source dataset, which is the sense in which Section 3.8 describes them as uncalibrated. Readers could adjust brightness, contrast and magnification as they would at a clinical workstation.

The task was deliberately matched to what the model’s kappa measures. The model is scored on matched lesions, i.e., on grading conditional on successful localization, so the readers were given the lesion location and asked only for the grade. Reader agreement with the reference standard, pairwise agreement between readers, and the model’s agreement computed on the same lesions are reported in Section 3.8, each with a cluster bootstrap interval.

## 3. Results

### 3.1. Effect of Lesion-Preserving Tiling

**Figure 7:**
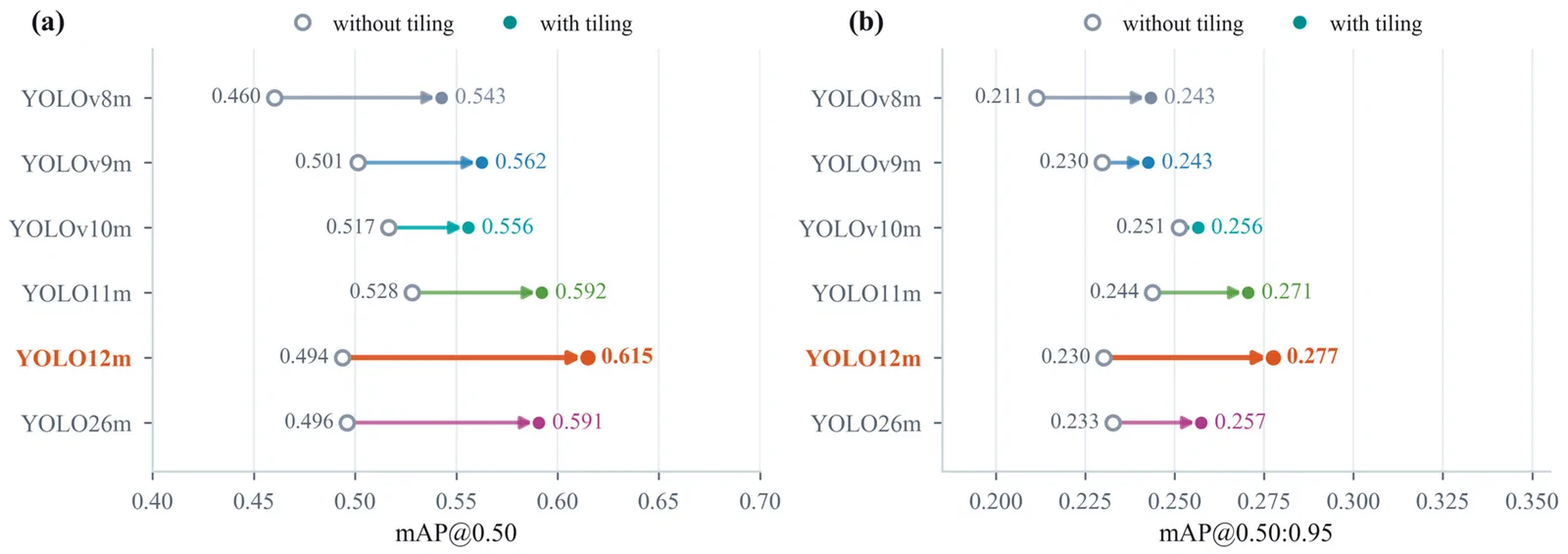
Effect of lesion-preserving tiling on six YOLO generations on the hold-out test set. Each row contrasts a generation trained on full down-sampled radiographs (open marker) with the same generation trained on lesion-preserving tiles (filled marker), for (**a**) mAP@0.50 and (**b**) mAP averaged over IoU 0.50 to 0.95. The arrow length is the gain attributable to tiling, which is positive for every generation.

Table 4 and Figure 7 contrast each generation trained on full down-sampled radiographs with the same generation trained on lesion-preserving tiles. Tiling raised mAP@0.50 for every architecture, by 0.039 to 0.121 with a mean of 0.077. Because both pipelines are scored on the same 561 radiographs, the gain can be tested directly with a paired radiograph-level bootstrap, and it is resolvable for five of the six generations: YOLOv8m +0.083 [+0.039, +0.124], YOLOv9m +0.061 [+0.018, +0.106], YOLO11m +0.064 [+0.021, +0.110], YOLO12m +0.121 [+0.074, +0.162] and YOLO26m +0.095 [+0.049, +0.139]. The exception is YOLOv10m, where the gain of +0.039 [−0.004, +0.081] falls just short of resolution while pointing in the same direction. The effect therefore follows from the representation rather than from any one backbone, and it is largest for YOLO12m, which rose from 0.494 to 0.615 at mAP@0.50 and from 0.230 to 0.277 at mAP@0.50:0.95.

Tiling also reorders the generations. YOLO12m gains the most and climbs from near the bottom of the down-sampled ranking to the top once tiled, whereas YOLOv10m moves the other way, so a backbone chosen on down-sampled images can be the wrong one. The improvement is larger at mAP@0.50 than at mAP@0.50:0.95, the signature of a resolution effect that recovers lesions previously missed rather than one that only tightens boxes already found.

### 3.2. Cross-Generation YOLO Benchmark

Table 5 reports the full ordinal suite for the six generations on tiles. YOLO12m attained the highest point estimates for localization, at mAP@0.50 0.615 and mAP 0.277, together with the highest severe-grade sensitivity at PAI 5 recall 0.602, and was selected as the backbone. The differences that underlie that ranking are, however, small relative to what this test set can resolve, and we therefore state what survives testing rather than the ranking alone. On a paired radiograph-level bootstrap, YOLO12m is not distinguishable from YOLO11m on any measure, the closest being mAP@0.50 at +0.023 with a 95% interval of [−0.024, +0.062] and a two-sided bootstrap p of 0.34, and it is not distinguishable from YOLO26m on localization, at +0.024 [−0.009, +0.056] with p equal to 0.18. It does differ from YOLO26m on severity grading, with the quadratic weighted kappa +0.039 [+0.006, +0.074] and PAI 5 recall +0.121 [+0.044, +0.200].

Release order did not predict accuracy. YOLOv10m, despite its NMS-free formulation, ranked below the older YOLOv9m at mAP@0.50, and YOLO26m, the most recent architecture, did not surpass YOLO12m on severity grading, so general-purpose architectural gains do not transfer automatically to small, low-contrast, long-tailed findings. Model size does not explain the ordering either: YOLO12m carries 20.2 million parameters against 25.9 million for YOLOv8m and 21.9 million for YOLO26m (Table 2). The property that does separate the generations reliably is stability. Across repeated training runs on different training and validation partitions, all scored on the same locked test set, mAP@0.50 was 0.610 with a range of 0.012 for YOLO12m against 0.605 with a range of 0.029 for YOLO26m and 0.570 with a range of 0.051 for YOLO11m. YOLO12m was therefore selected as the most stable of the three most recent generations and the strongest on severity-weighted precision and severe-grade recall, and not on the basis of a localization advantage the data do not support.

The catastrophic error rate does not separate the generations either. Across the six, it spans four to seven lesions of 985, which is 0.41% to 0.71%, and YOLO12m sits at six rather than at the minimum, so on this measure it is not the best of the six. No pairwise difference among the six survives the bootstrap, since an interval built on counts of this size spans zero in every case, and we therefore treat the catastrophic column of Table 5 as uninformative for ranking.

### 3.3. Cumulative Contribution Ablation

Starting from the tiled YOLO12m baseline, the components were added cumulatively (Table 6). The ordinal loss of Equation (4) raised Sev-wAP from 0.667 to 0.691, a paired difference of +0.024 with a 95% interval of [+0.002, +0.049], while leaving mAP@0.50 essentially unchanged at 0.609, reorganizing the errors rather than improving detection, as Equation (2) intends. It also lowered the catastrophic rate from 0.61% to 0.41%, although that change is six lesions against four and is not resolvable on this test set, with a difference interval of [−0.72, +0.30] percentage points. The P2 head then raised mAP@0.50:0.95 from 0.279 to 0.298 and brought the catastrophic count to its minimum of two lesions in 985.

**Figure 8:**
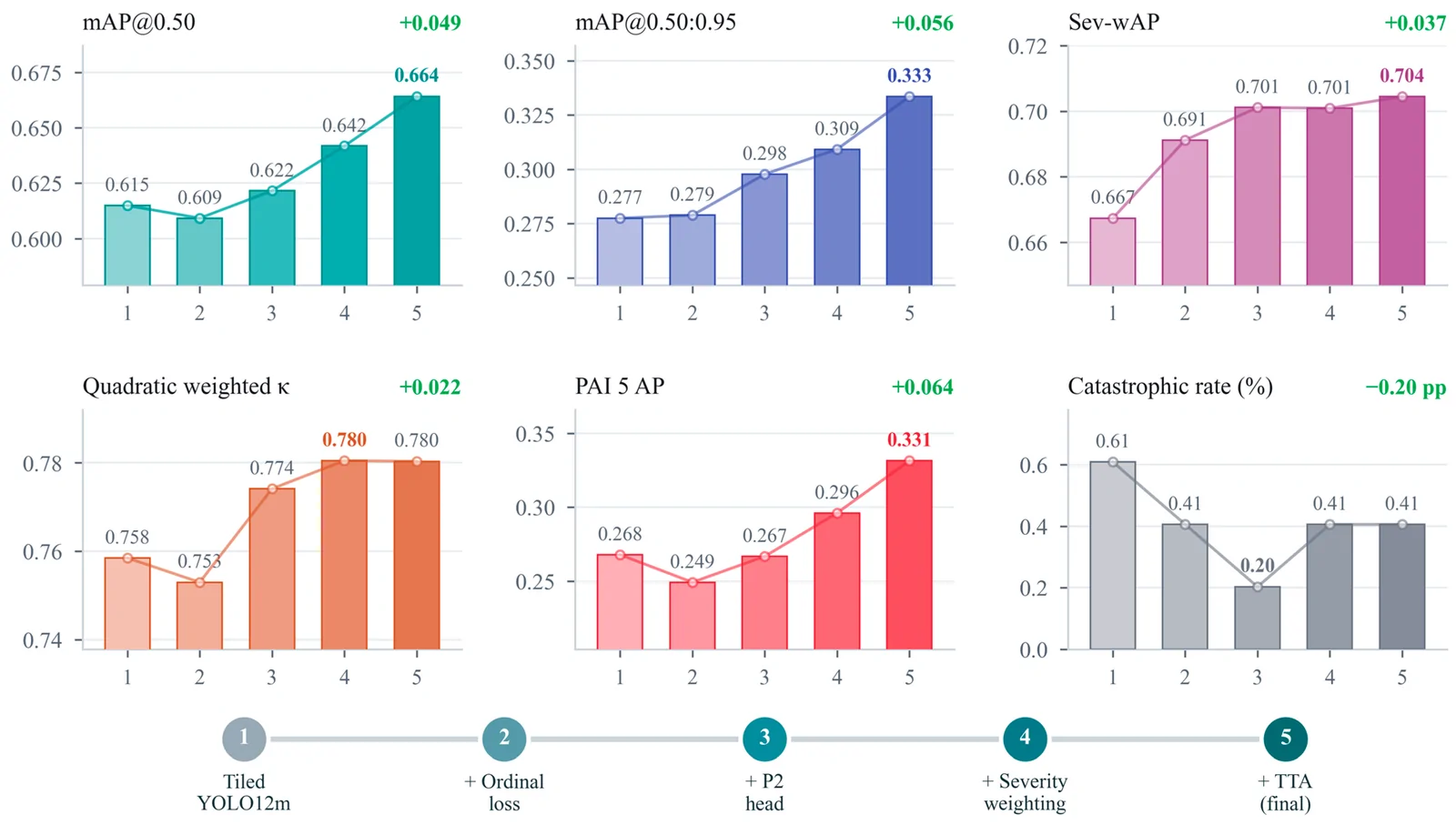
Cumulative contribution of the proposed components on YOLO12m, evaluated on the hold-out test set. Each panel carries one metric on its own scale and the five bars are the cumulative configurations identified in the ribbon beneath, in which every step retains all components to its left. The badge above each panel gives the net change from the tiled baseline to the final model.

Severity weighting traded part of that margin for sensitivity, returning the catastrophic count to four lesions while raising PAI 5 recall from 0.542 to 0.614 and PAI 5 average precision from 0.267 to 0.296. The trade is clinically the preferable direction, since a severe lesion reported as established is still examined whereas one not reported is not. Test-time augmentation added the last increment without retraining, carrying mAP@0.50 to 0.664, mAP@0.50:0.95 to 0.333 and PAI 5 recall to 0.663. Figure 8 collects the five configurations metric by metric. The final configuration itself carries the intervals a reader needs in order to judge its precision: mAP@0.50 0.664 [0.626, 0.705], Sev-wAP 0.704 [0.676, 0.733], a quadratic weighted kappa of 0.780 [0.740, 0.816], PAI 5 recall 0.663 [0.559, 0.760] and a catastrophic rate of 0.41% [0.10, 0.83], each from the same radiograph-level cluster bootstrap.

Two points should be read alongside the table. First, only the cumulative gain and the test-time augmentation step are individually significant: the full framework improves on the tiled baseline by +0.049 [+0.020, +0.080] in mAP@0.50 and +0.037 [+0.014, +0.062] in Sev-wAP, and test-time augmentation contributes +0.022 [+0.007, +0.039], whereas on mAP@0.50 no other single step is resolvable in isolation. The ordinal loss is resolvable on the axis it was designed to move, raising the severity-weighted precision by +0.024 [+0.002, +0.049], as Section 3.9 reports. Second, test-time augmentation was also run on the tiled baseline, where it produces a comparable increment of +0.015 in mAP@0.50, so the final row reflects an essentially additive contribution rather than an interaction with severity weighting.

Both hyperparameters introduced in this work were swept rather than assumed, and Table 7 reports the sweep. The smoothing coefficient was varied with the P2 head held out, and the severity weights were varied on top of the ordinal loss and the P2 head, so each was read against the configuration in which it acts. A coefficient of 0.10 spreads too much mass onto the neighbouring grades and degrades every metric, and inverse-frequency weighting suppresses the majority grade far enough to cost average precision, which is why the milder setting was retained.

### 3.4. Training Dynamics

**Figure 9:**
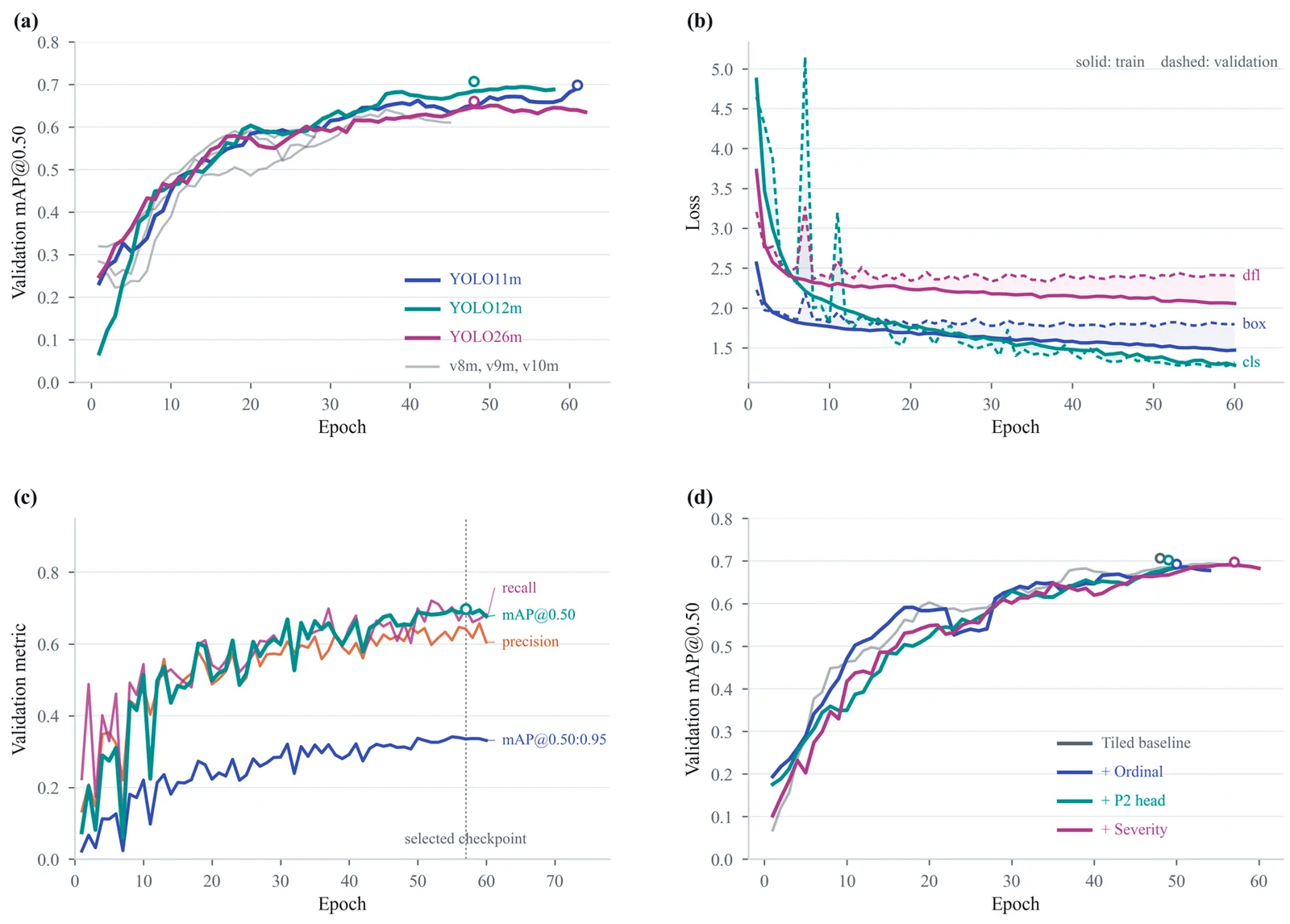
Training dynamics of the models reported above, read from the per-epoch logs written during training. (**a**) Validation mAP@0.50 of the six YOLO generations on lesion-preserving tiles, with the three most recent generations drawn in colour and the earlier three held in grey, and the circle marking the best epoch of each run. (**b**) Loss components of the final model, with the solid line the training term, the dashed line its validation counterpart and the shaded band the gap between them. (**c**) Validation mAP@0.50, mAP@0.50:0.95, precision and recall of the final model, with the dotted rule at the selected checkpoint. (**d**) Validation mAP@0.50 of the successive ablation configurations, each shown against the tiled baseline held in grey, with circles again marking the best epoch of each run. Runs terminate at different epochs because early stopping was applied to every model under an identical criterion.

Figure 9 collects the training behaviour of every model reported above, read from the per-epoch logs written during training. Figure 9a shows that the six generations follow broadly similar trajectories on tiles and terminate at different epochs under an identical early-stopping criterion, so the benchmark of Table 5 compares converged runs rather than runs cut short at a common epoch count. Figure 9b,c describe the final model. All three loss components fall smoothly and none of the validation curves turns upward, the box and distribution-focal terms flattening onto a plateau while the classification term is still falling at the selected checkpoint, so the added P2 capacity does not overfit, even though the gap to training widens as the training loss keeps decreasing. The classification gap is the smallest of the three at 0.035, so the ordinal-smoothed severity-weighted target is no harder to fit than the nominal one, and in Figure 9c precision and recall rise together, with the retained checkpoint the one attaining the highest validation mAP@0.50.

Figure 9d places the ablation configurations on a common validation axis. The best validation values do not increase monotonically along the ladder, so the components do not make the detector fit the validation data better. They change the objective being fitted, and their benefit therefore appears in the ordinal and per-grade test metrics rather than in validation mAP.

### 3.5. Per-Grade and Severity Analysis

**Figure 10:**
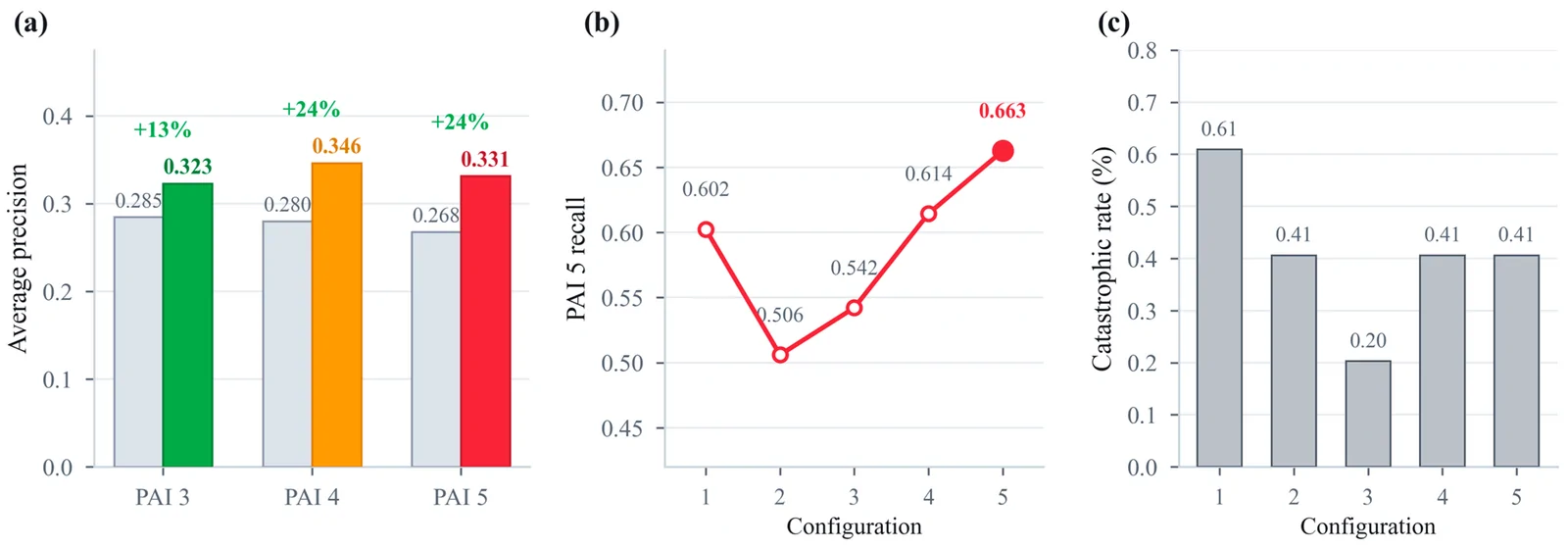
Where the improvement lands. (**a**) Average precision per grade for the tiled baseline, shown in grey, against the final model, with each grade drawn in its severity colour (green for PAI 3, orange for PAI 4, red for PAI 5) and the relative gain above each pair. (**b**) Recall of the severe PAI 5 grade across the five cumulative configurations, which follow the rows of Table 6 from the tiled baseline to the final model. (**c**) Catastrophic error rate across the same configurations. The severe grade, which is both the rarest and the most consequential, benefits most.

Figure 10 decomposes the improvement by grade. Average precision rose for all three grades, by 13% for PAI 3 and by 24% for both PAI 4 and PAI 5, so the severe-grade gains do not come at the expense of the majority class. The three grades converged to within 0.023 of one another, PAI 4 highest at 0.346, PAI 5 next at 0.331 and PAI 3 lowest at 0.323, so the severe grade is no longer the hardest, as it was on the tiled baseline. PAI 5 recall climbed from 0.602 to 0.663, the severity weighting contributing the largest single step of 0.072 and test-time augmentation a further 0.049, while the catastrophic rate stayed at or below 0.61% throughout.

**Figure 11:**
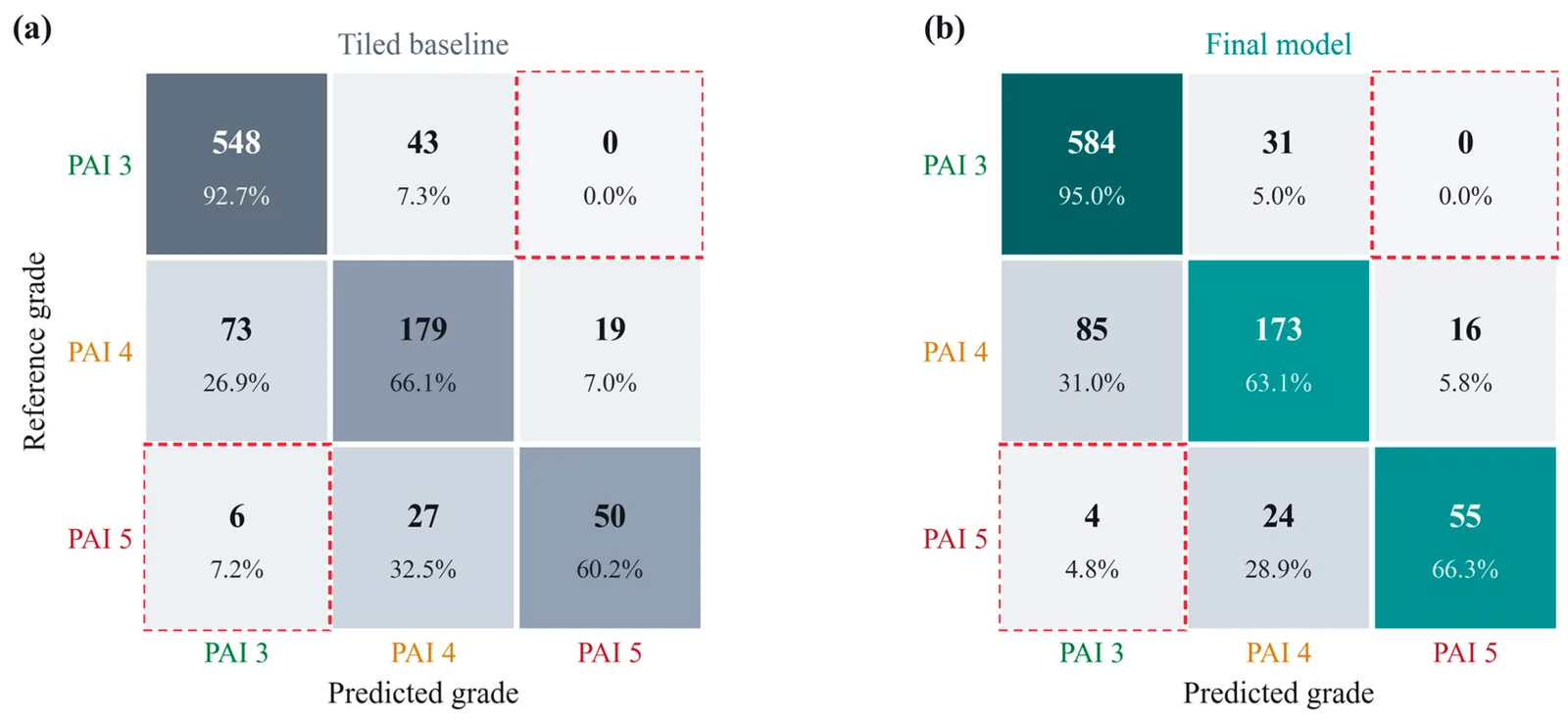
Confusion matrices of matched lesions for (**a**) the tiled baseline and (**b**) the final model, giving the lesion count with the row-wise percentage beneath. Diagonal cells are shaded on the accent ramp and off-diagonal cells on a neutral ramp, so correct and incorrect mass are separable at a glance. The dashed corners mark catastrophic confusions between the mildest and the severest grade.

**Figure 12:**
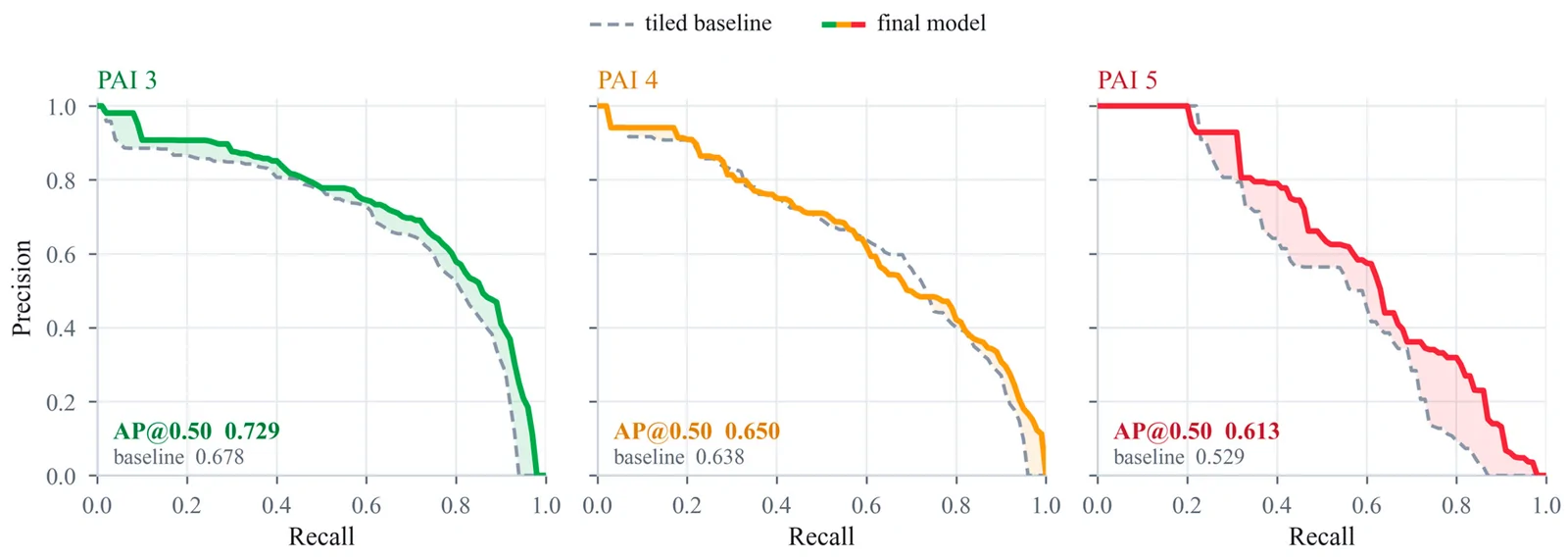
Precision–recall behaviour per grade at IoU 0.50, comparing the tiled baseline with the final model, whose curve is drawn in the colour of its grade. The shaded area is the precision gained by the proposed framework where it exceeds the baseline. Values are the 101-point interpolated AP@0.50, whose mean over the three grades reproduces the mAP@0.50 reported in Table 6.

**Figure 13:**
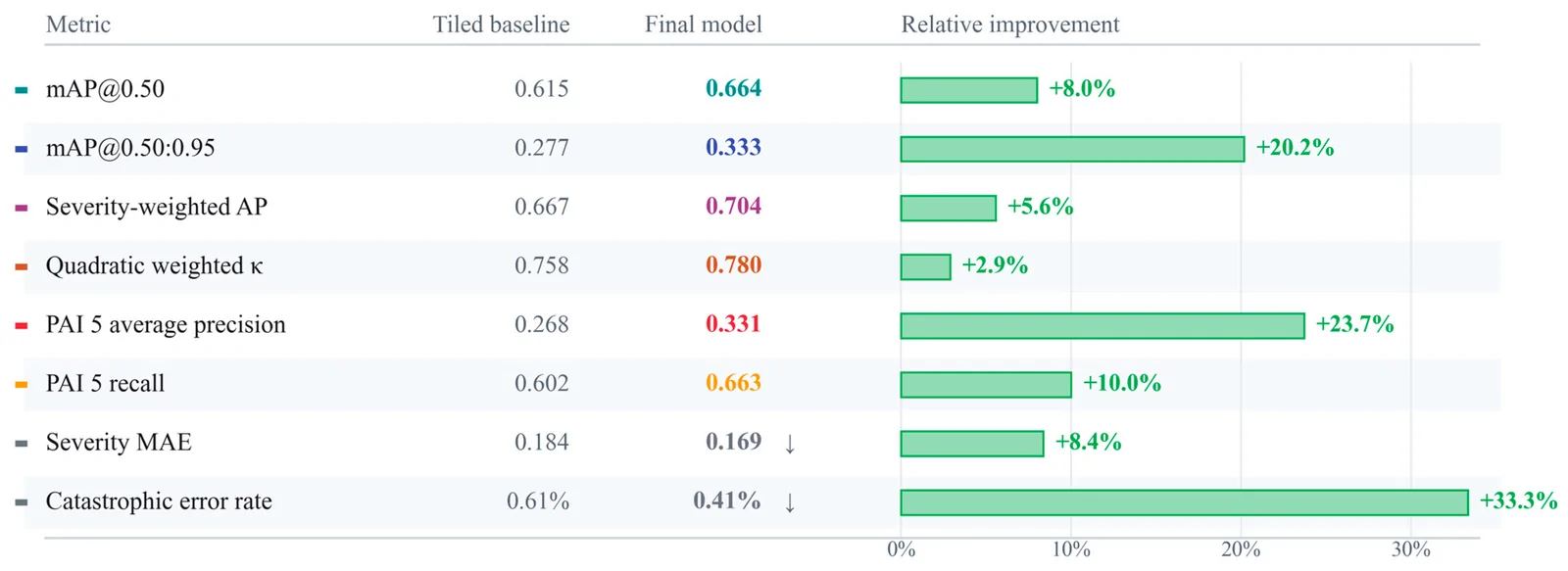
Summary of the tiled baseline against the final model on every headline metric. The numeric columns give the exact values and the bars the relative change, oriented so that an improvement is always positive. The final two rows are error measures, for which a decrease is the improvement. Every metric improves simultaneously. ↓ decrease.

The confusion matrices of Figure 11 and the precision–recall curves of Figure 12 show the same shift. Correct PAI 3 classification rose from 548 to 584 lesions and correct PAI 5 from 50 to 55, and the catastrophic confusions between the two extremes fell from six to four. The four that remain are all severe lesions graded as mild rather than the reverse, the residual error the severity components reduce, but do not yet remove. PAI 4 is the one grade whose diagonal share does not rise, and the movement is entirely into the neighbouring PAI 3 cell, so the residual disagreement stays one grade wide. PAI 5 gained the most area, its interpolated AP@0.50 rising from 0.529 to 0.613, spread across the mid and high recall range and peaking near 0.8 recall. Severity MAE fell from 0.184 to 0.169 and the quadratic weighted kappa rose from 0.758 to 0.780, both consistent with errors confined to the immediate ordinal diagonal. Figure 13 collects the comparison on every headline metric, all of which move in the improving direction at once.

### 3.6. Qualitative Results

**Figure 14:**
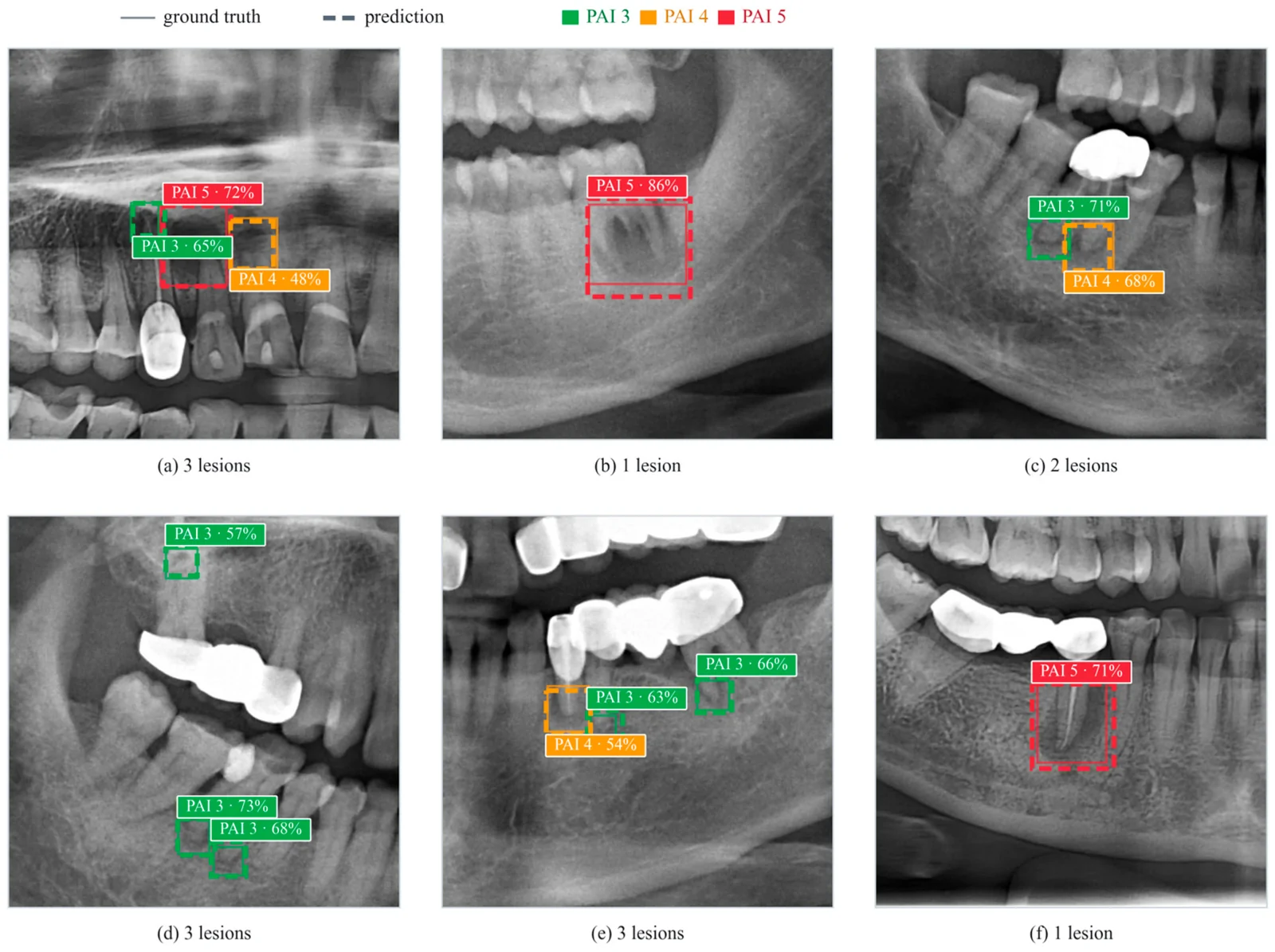
Qualitative results of the final model on hold-out tiles, selected to span the three grades and a range of lesion counts encountered in the test set. Reference annotations are drawn as thin solid outlines and model predictions as heavier dashed outlines carrying the predicted grade and its confidence. Boxes are shown at a confidence of 0.30, a display threshold used only for these panels since the reported metrics integrate over all confidences. In every panel shown, all lesions are detected, all are graded correctly and no spurious box is produced. (**a**) carries all three grades within a single tile and (**b**) is the highest-confidence severe detection in the test set.

Figure 14 shows final-model detections on hold-out tiles that span the three grades and carry one, two or three lesions. Every lesion is detected and correctly graded with no spurious box at the display confidence of 0.30. The panels include a tile carrying all three grades, three early PAI 3 lesions one of which the down-sampled baseline misses on the full radiograph, and the highest-confidence severe detection in the test set at 86%.

**Figure 15:**
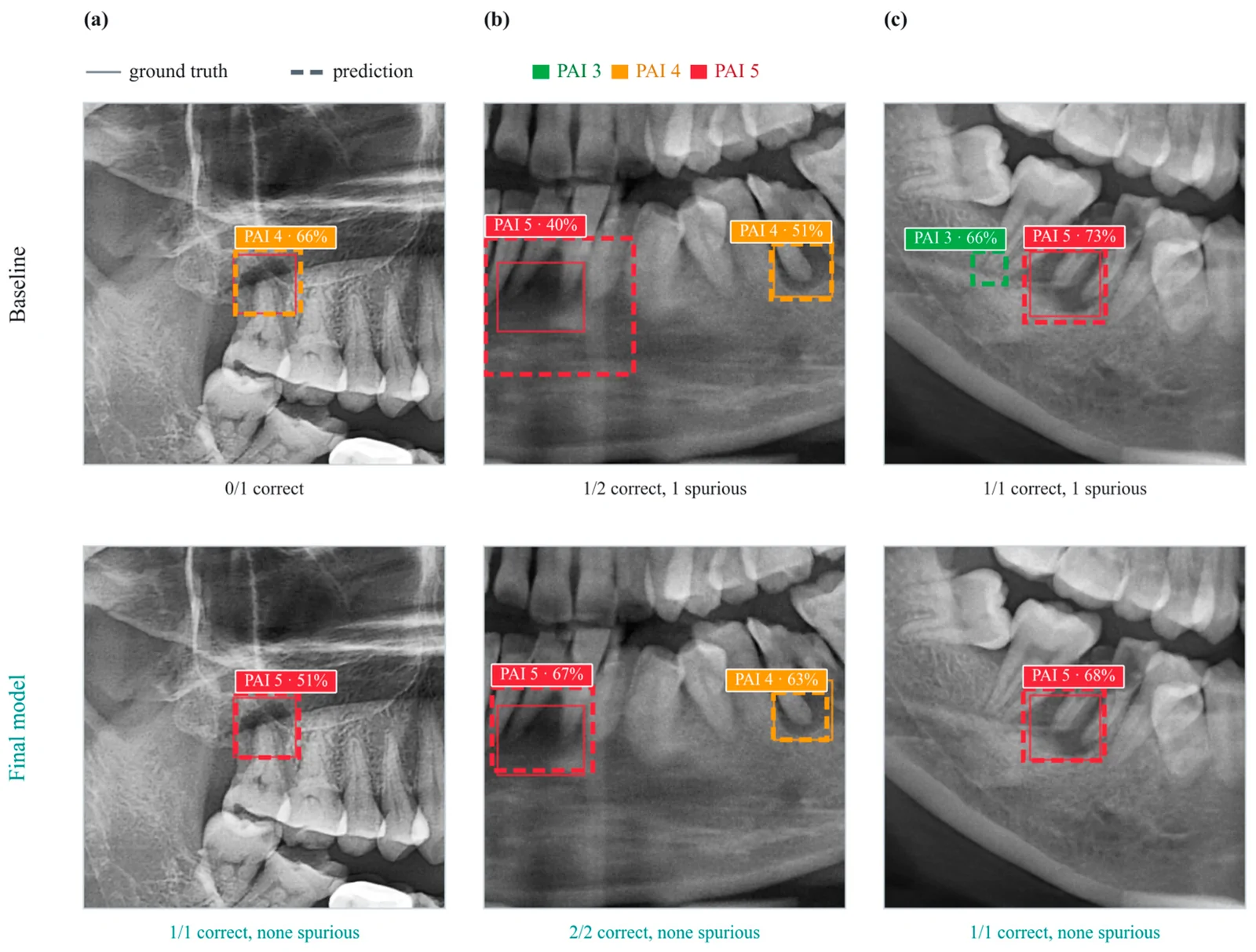
Tiled baseline (top row) against the final model (bottom row) on the same three hold-out tiles. In (**a**), the baseline localizes the lesion, but assigns the neighbouring grade, which the final model corrects to the reference grade of PAI 5. In (**b**), the baseline’s severe detection falls below the matching threshold, counting as both a missed lesion and a false positive, while the final model recovers both lesions cleanly. In (**c**), the baseline adds a spurious mild detection that the final model does not produce. Counts beneath each panel are obtained by the same IoU 0.50 matching used throughout the evaluation.

**Figure 16:**
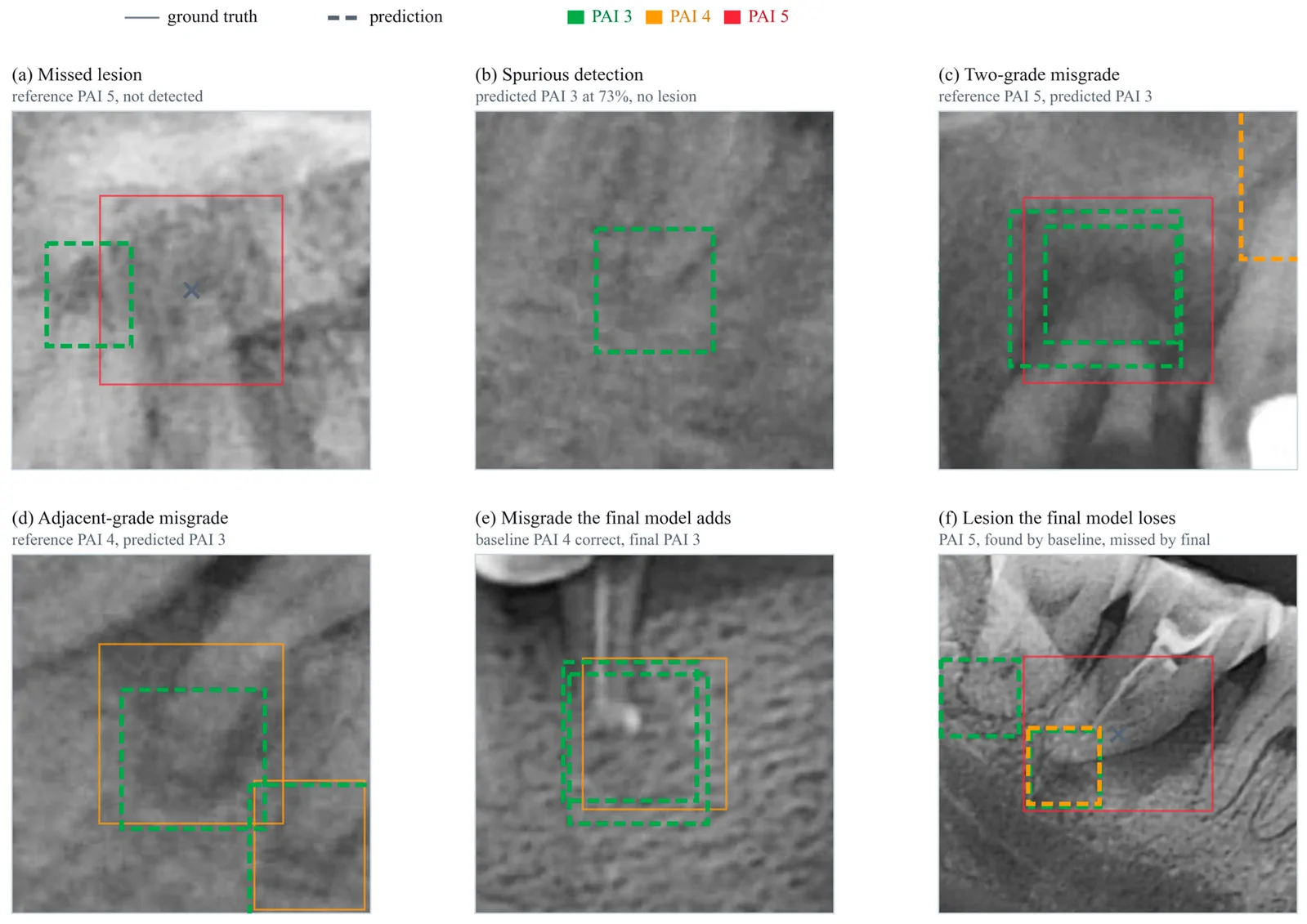
Failure cases of the final model on hold-out tiles, one panel for each error mode the evaluation counts. (**a**) A reference lesion the model does not detect, its location marked with a cross. (**b**) The highest-confidence spurious detection, on anatomy carrying no annotated lesion. (**c**) One of the four two-grade misgrades remaining in the test set. (**d**) The highest-confidence adjacent-grade misgrade. (**e**) A lesion the tiled baseline grades correctly and the final model does not. (**f**) A lesion the tiled baseline detects and the final model misses. Reference annotations are drawn as thin solid outlines and predictions as heavier dashed outlines in the colour of their grade. Cases are enumerated by the same IoU 0.50 matching and 0.30 display threshold used throughout and chosen by a stated rule for each mode rather than by hand, and (**e**,**f**) are errors the proposed components introduce relative to the baseline rather than errors they repair.

These single cases illustrate error types that Table 6 quantifies in aggregate. Figure 15 repeats three hold-out tiles for the tiled baseline and the final model in turn, so the change the components make can be read on the same material. Because both of those figures show the framework working, and one of them shows only errors it repairs, they give no sense of how the model fails. Figure 16 therefore presents failure cases exclusively, enumerated by the same IoU 0.50 matching and 0.30 display threshold used throughout and selected by a stated rule rather than by hand. At that operating point, the final model misses 173 of the 985 reference lesions, produces 497 spurious detections, which is 0.89 per radiograph, and misgrades 132 of the lesions it matches. Relative to the tiled baseline, it also introduces errors of its own: 30 lesions that the baseline graded correctly it does not, and 40 lesions that the baseline found it misses, 5 of them PAI 5 lesions.

**Figure 17:**
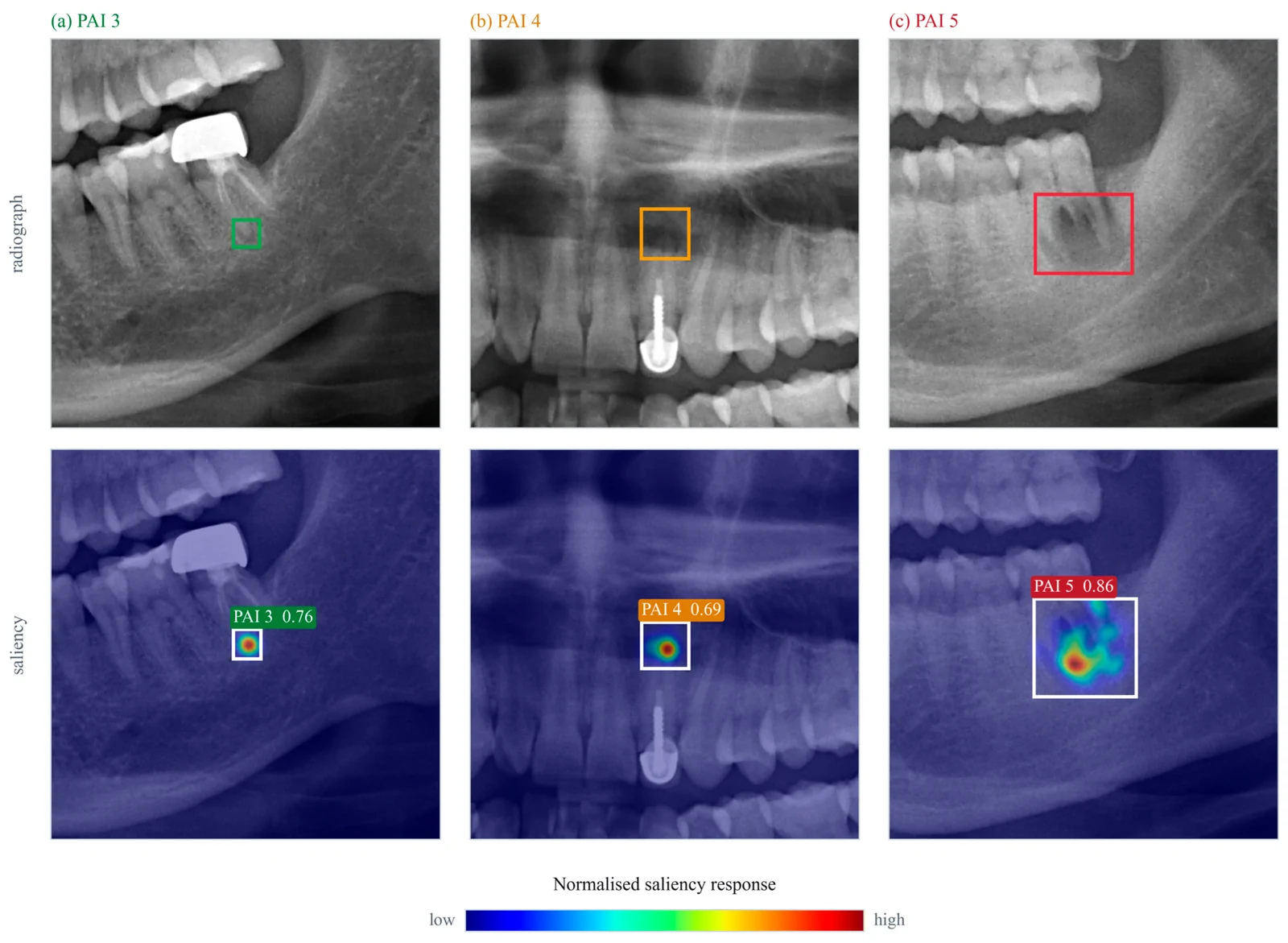
Saliency of the stride-4 P2 level that feeds the detection head, for one correctly detected and correctly graded lesion of each grade: (**a**) PAI 3, (**b**) PAI 4 and (**c**) PAI 5. The upper row is the radiograph with the reference annotation outlined in the colour of its grade, and the lower row the same field with the saliency map overlaid and the model prediction drawn in white with its confidence. The map is the element-wise product of the P2 activation and its gradient with respect to the summed class score of every anchor inside the reference lesion, read before non-maximum suppression, normalised to its maximum and smoothed for display only.

Figure 17 addresses what drives the predictions. For one correctly detected lesion of each grade, it shows the radiograph and the response of the stride-4 P2 level that feeds the detection head, computed as the element-wise product of activation and gradient with the target taken as the summed class score of every anchor inside the reference lesion, read before non-maximum suppression so that no gradient passes through it. We quantify the maps rather than leave them to the eye: 100%, 97% and 90% of the total response falls inside the reference lesion for the mild, established and severe example, although the lesion occupies only 0.5%, 1.6% and 5.0% of the tile. The departure from the original Grad-CAM formulation is deliberate. Weighting the activation map by globally average-pooled gradients assumes a class score derived from globally pooled features, which does not hold for a dense detection head whose target score belongs to one spatial location, and on these tiles it produced diffuse maps responding to trabecular texture and image borders with 5% or less of the response inside the annotation.

### 3.7. Label-Free Inference Results

Every result above is obtained on tiles whose placement used the reference annotations. Table 8 reports the framework under the label-free pipeline of Section 2.10, in which no annotation is used at any point and the unit of evaluation is the radiograph. On the locked test set, the pipeline reaches mAP@0.50 0.5125, mAP 0.2203, Sev-wAP 0.5250 and a quadratic weighted kappa of 0.7654, against mAP@0.50 0.4937 for a detector applied to the down-sampled full radiograph. The comparison against that baseline is mixed rather than uniform. Localization is not distinguishable, the paired difference in mAP@0.50 being +0.019 with a 95% interval of [−0.022, +0.059]. Grade agreement is resolvably better, the weighted kappa differing by +0.038 [+0.001, +0.077]. Severity-weighted precision is resolvably worse, at −0.037 [−0.065, −0.008], which follows from the false positives. A dense scan of the whole radiograph necessarily produces on normal anatomy that the down-sampled single pass never examines at this resolution. Catastrophic confusion is identical at 0.20% for both. We therefore describe the label-free pipeline as comparable to the full-image baseline rather than superior to it, with the trade-off lying between grade agreement and precision rather than in localization.

The mismatch set out in Section 2.10, between a detector that never sees a pure-background window during training and a dense scan in which most windows contain no lesion, proved measurable: of all detections the originally trained model produced above a confidence of 0.30 during a dense scan of the validation radiographs, 41.2 per cent arose in windows containing no lesion whatsoever. The same model behaves the same way on the test radiographs, where the share is 37.5 per cent and label-free mAP@0.50 reaches only 0.373, well below the full-image baseline, as the second row of Table 8 reports. Retraining the identical configuration with lesion-free tiles added, sampled from the same dense grid used at inference and drawn only from training radiographs, reduced that share to 12.6 per cent on validation and 9.6 per cent on testing, and raised label-free mAP@0.50 to 0.4925 on validation and 0.5125 on testing.

The correction is not free. On the annotation-driven tiles, the background-trained variant is weaker than the model of Table 6, at mAP@0.50 0.594 against 0.642 and Sev-wAP 0.646 against 0.701, and its PAI 5 recall falls from 0.614 to 0.494, since half of its gradient updates now concern background rather than lesions. We therefore retain the model of Table 6 as the model of this study and present the background-trained variant as a separate analysis of deployment behaviour. The wider point is a design lesson for lesion-preserving tiling schemes in general: a scheme that emits tiles only where lesions are found produces a detector that has never been asked to reject normal anatomy, and that deficit is invisible to any evaluation conducted on the same lesion-centred tiles.

### 3.8. Reader Study Results

Table 9 reports the reader study of Section 2.11. Scored against the same reference standard on the same stratified sample, the three endodontists achieved quadratic weighted kappa of 0.704 [0.628, 0.773], 0.572 [0.493, 0.649] and 0.525 [0.441, 0.605], with exact agreement of 66.3%, 56.2% and 49.8%. Agreement between the readers themselves was lower still, at 0.648 for the first pair, 0.509 for the second and 0.312 for the third, and the two-grade disagreements that the framework treats as catastrophic occur between readers as well, in 1.61%, 8.43% and 17.27% of the sample for the three pairs against 1.61% for the framework against the reference standard. The model was evaluated on the same lesions and achieved 0.766 [0.700, 0.822] with exact agreement of 73.0%.

Averaged over the three pairs, agreement between the readers is 0.490 in quadratic weighted kappa. We report pairwise weighted coefficients because the quadratic weights respect the ordinal scale and because the spread between pairs is itself informative. Weighted multi-rater coefficients also exist and would summarize the three readers in a single figure, and the mean of the three pairwise values serves that purpose here, with the individual pairs spanning 0.312 to 0.648.

Two readings follow. The first is that the premise of this work is confirmed on its own material rather than taken from the literature: severity grading of periapical lesions on panoramic radiographs is markedly observer-dependent, with expert pairwise agreement spanning 0.312 to 0.648 on identical images with the lesion already localized. The second concerns what the model’s higher figure means, and here we are deliberately restrictive. The model was trained on 4623 lesions annotated under this reference standard and has therefore learned the annotators’ convention, whereas the readers were not calibrated to it. The correct statement is that the framework reproduces the reference convention more consistently than uncalibrated expert readers, which supports a claim about reproducibility.

### 3.9. Comparison of Ordinal Formulations

The ordinal loss of Section 2.4 acts by softening the target. An equally established alternative acts on the loss instead, leaving the target one-hot and scaling each class term by one plus the squared ordinal distance from the true grade, with the scale factor set to one, so that the term for an adjacent grade is weighted twice and the term for a two-grade error five times the term for the correct grade, which makes a two-grade error two and a half times as costly as an adjacent one. Because this optimizes directly what the quadratic weighted kappa penalizes and because it leaves the architecture, the suppression stage and the decoding untouched, it is a controlled comparator rather than a confounded one. We trained it under the identical pipeline, partition and schedule, and Table 10 reports both against a nominal objective.

Neither formulation dominates, and we report the comparison in full, including where it does not favour the one we adopted. In isolation, the distance-weighted loss attains higher severe-grade recall than the target smoothing, 0.614 against 0.506, and this is the only difference between the two that is resolvable on this test set, at +0.108 [+0.021, +0.200]. It also reaches localization comparable to the full three-component configuration, at mAP@0.50 0.6425 against 0.6418, a difference of +0.0007 [−0.027, +0.031]. The target smoothing, for its part, is the only one of the two that resolvably improves the severity-weighted average precision over a nominal objective, at +0.024 [+0.002, +0.049], and the full configuration remains ahead on the severity-aware measures, with mAP 0.309 against 0.297, Sev-wAP 0.701 against 0.683 and a weighted kappa of 0.780 against 0.774.

The two formulations therefore trade severe-grade sensitivity against severity-weighted precision, and no further difference between them is resolvable here. We do not claim that the smoothing is the optimal ordinal objective for this task. It is one competitive formulation among several, and its weakness in isolation, a loss of severe-grade recall, is precisely what the severity weighting of Section 2.6 exists to recover, as the cumulative table shows with PAI 5 recall moving from 0.506 to 0.542 with the addition of the P2 head and to 0.614 with severity weighting.

### 3.10. Calibration and Operating Behaviour

Sweeping the confidence threshold traces the free-response characteristic of the final model: at 0.10 it localizes 93.5% of the reference lesions at 2.46 false positives per radiograph, at 0.30 it localizes 82.4% at 0.89 false positives per radiograph, and at 0.50 it localizes 63.4% at 0.21. The tiled baseline is below it throughout, at 87.0%, 73.1% and 44.7% for the same three thresholds with 1.54, 0.49 and 0.10 false positives per radiograph, so the proposed components buy sensitivity at the cost of additional false positives at every threshold rather than at one operating point only.

Calibration is the weaker property. The expected calibration error of the final model is 0.318, improved from 0.382 for the tiled baseline but still large, which means the confidence a detection carries should be read as a ranking score rather than as the probability that its grade is correct. This is the usual behaviour of a one-stage detector trained with a classification loss that is not calibrated, and it bears on deployment: a threshold chosen on this scale selects an operating point, it does not select a confidence level.

## 4. Discussion

This work improved the detection and, above all, the ordinal severity grading of periapical lesions on panoramic radiographs. Three findings stand out. Lesion-preserving tiling is a prerequisite for small-lesion detection, raising mAP@0.50 for every generation and by more than twelve points for YOLO12m. Among six generations under one protocol YOLO12m was the most stable across repeated runs and the strongest on severe-grade sensitivity, and the newer YOLO26 did not surpass it on any measure. Treating severity as ordinal changed which errors the detector makes rather than only how many, and combined with the high-resolution head and the severity weighting, it raised every headline metric of Table 6 at once.

The ranking of Table 5 is worth reading architecturally. The two generations that introduce explicit attention into the backbone, YOLO11 with its C2PSA module and YOLO12 with area attention and R-ELAN, take the first two places on tiles by point estimate, and take them on mAP@0.50, on the severity-weighted average precision, on the weighted kappa and on severe-grade recall alike, whereas the two that replace non-maximum suppression with an end-to-end head, YOLOv10 and YOLO26, do not. Attention over spatial regions plausibly helps because a periapical radiolucency is defined by a low-contrast change against trabecular bone rather than by a strong edge, so features that pool context around a root apex separate lesion from background better than features computed locally. Removing non-maximum suppression, by contrast, simplifies inference without changing how small low-contrast targets are represented, which is consistent with the absence of a gain here. This is an observation on one dataset and one task rather than a general ranking of the architectures.

The components are best judged by the error structure of Figure 11. Confusion concentrates on the immediate diagonal, adjacent-grade errors between PAI 3 and PAI 4 and between PAI 4 and PAI 5, accounting for almost all of it, while off-by-two PAI 3 versus PAI 5 errors stay rare. This is expected, since the PAI is an ordinal approximation of a continuous process: periapical rarefaction progresses without discrete steps, the boundary between adjacent grades is inherently fuzzy, and the model hesitates between neighbours, much as an examiner does over a borderline lesion. Agreement between observers on two-dimensional radiographs is itself limited, and the intermediate grades, which share their radiographic appearance with the grades on either side, have been reported as the hardest to score automatically as well.

Much of the residual confusion follows from the modality itself. Panoramic projection attenuates precisely the margin and density cues that PAI scoring depends on, and Section 4.3 sets out how and why that bears on the scope of the framework. The consequence for the error structure is specific enough to state here. Because loss of lamina dura continuity is the pivotal cue separating an intact from an established periapex, a modality that renders it ambiguous preferentially produces PAI 3 versus PAI 4 uncertainty, and that is the axis on which the model errs most: it carries 116 of the 160 residual disagreements.

PAI 4 is the hardest grade because, as the intermediate one, it is flanked by two decision boundaries and inherits ambiguity from both. Its defining judgement, whether a radiolucency is a well-defined area of destruction rather than the diffuse rarefaction of PAI 3 or the expansile lesion of PAI 5, rests on an appraisal of margin and extent that even calibrated examiners apply inconsistently, which explains its spread across all three predicted columns and its tendency to be under-graded to PAI 3.

What remains on the severe grade reflects data scarcity together with anatomical camouflage. Severe lesions are the rarest of the three and an extensive radiolucency can blend into the sinus or marrow spaces, extend beyond the focal trough where it loses its density gradient, or present deceptively defined margins that invite a conservative PAI 4 call. The references that no detection matches at all are mild lesions rather than severe ones, genuine lucencies dismissed as the mental or incisive foramen, nutrient canals, marrow spaces or tooth crypts, which are also the most likely source of false positives, errors experienced clinicians make as well.

**Figure 18:**
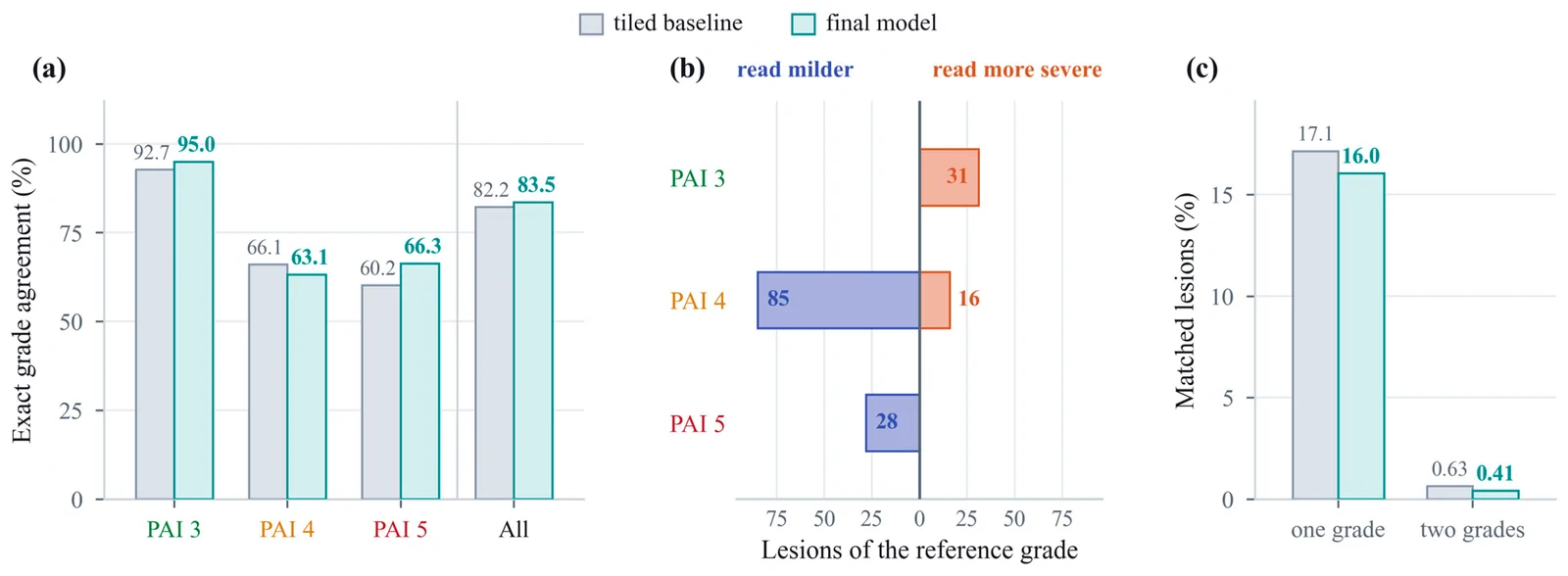
Anatomy of the residual disagreement, computed from the confusion matrices of Figure 11. (**a**) Exact grade agreement per reference grade and over all matched lesions for the tiled baseline against the final model. (**b**) Direction of the residual disagreement of the final model, with the lesion counts of each reference grade read as milder placed left of the axis and read as more severe placed right of it. (**c**) Share of matched lesions whose predicted grade lies one grade and two grades from the reference for the same two models. Agreement improves for the mild and the severe grade, and overall the disagreement that remains is almost entirely one grade wide and the share spanning the full scale falls from 0.63% to 0.41% of matched lesions.

The overall structure is clinically favourable, and Figure 18 decomposes it. Exact agreement rose for the mild and the severe grade, from 92.7% to 95.0% and from 60.2% to 66.3%, and fell for the intermediate one, from 66.1% to 63.1%, so PAI 4 absorbed the boundary shift that sharpened the other two. The direction of the residual disagreement is not symmetric: PAI 4 is read as milder in 85 lesions against more severe in 16, and against the baseline its under-grading to PAI 3 rose from 73 to 85, the same boundary shift seen from the other side. Over all matched lesions the profile still moved the right way, exact agreement rising from 82.2% to 83.5% and disagreement spanning the full scale falling from 0.63% to 0.41% of matched lesions, which is 0.61% to 0.41% when normalized over all reference lesions as Equation (11) defines it. That shift matters most under the cost structure this work assumes, in which the off-by-two error is the most consequential. This is a redistribution within the adjacent band rather than a new catastrophic error, and it leaves reliable separation of PAI 4 as the main target for future work.

### 4.1. Comparison with the Literature

Table 11 places the framework among two-dimensional radiograph studies, split into presence-only detection and severity grading. The comparison is indirect, as the studies differ in modality, unit of analysis, reference standard, and above all in what they predict. The presence-only block solves an easier problem: its highest entry, an mAP of 0.953 from Çelik et al. with RetinaNet [24], counts a detection correct whenever it localizes a lesion, whereas a detection here is correct only if it also assigns the right grade, so the two numbers are not commensurable. The block is nonetheless instructive about image handling. Fang et al. reached AP@0.50 0.842 on the largest panoramic benchmark, 3673 radiographs carrying 5662 lesions, yet their own average precision on small lesions stayed at 42.3 against 58.7 on large ones [43], and Mureșanu et al., resizing each radiograph to 1024 × 1024, reported 0.412 recall for the periapical class against 0.899 for root canal treatments and 0.964 for surgical devices [44], which is the motivation for native-resolution tiling.

The severity block is the substantive comparison. Moidu et al. scored the full five-point scale on 3000 root areas and predicted the correct grade for 56.11% of them, falling to 30% for PAI 5, the 86.3% accuracy they report following a collapse of the scale to healthy against diseased [30]. Viet et al. are the closest antecedent, from the group that released this dataset and using the same PAI 3-to-PAI 5 scheme, comparing Faster R-CNN with YOLOv4 on 2658 intraoral radiographs [32]. Saber et al. coupled three YOLO generations with the full scale on 699 intraoral radiographs at mAP@0.50 0.866 [33] and Erkal et al. derived severity from a lesion-area ratio, an ordered, but geometric proxy [34]. In all of these, the grades enter as unordered categories, an off-by-two misgrade penalized as an off-by-one. Lee et al. are the exception, classifying five grades with a state-space model and reporting a quadratic weighted kappa of 0.713 alongside 54.72% accuracy [31]. Their kappa is comparable in kind with the 0.780 here, though five grades include the confusable healthy and doubtful categories while intraoral classification avoids localization. Even there, ordinality enters only at evaluation, the training loss remaining nominal, and no study reports a severity MAE or a catastrophic-misgrade rate.

Two features of this literature bear on how its reported figures should be read, and they apply to the present study as much as to the work it is compared with. Most of these collections are enriched, in the sense that they are assembled around teeth or radiographs already known to carry a finding, and several are filtered by a reader consensus that removes the equivocal cases, so the accuracy they report describes performance on unambiguous disease rather than on a consecutive clinical series. Few are validated on material from a centre other than the one that produced the training data, so how far any of these figures transfers across populations, devices and acquisition protocols is largely unmeasured.

Two limits of this work follow. Neither ordinal learning inside a detector nor tiling is new. Reg R-CNN already replaces the classification head of a detector with a regression head so that grading preserves the ordinal relation between grades [45], and patch-based processing is routine wherever the target is small relative to the image. What is new is their conjunction for this task, a tiling rule that keeps every lesion whole and unduplicated at native resolution, a six-generation YOLO benchmark under one protocol and an ordinal objective with a matching metric suite, which together let the error structure of Figure 11 be reported as a clinical property with catastrophic confusion held at 0.41%.

### 4.2. Advantages of the Proposed Framework

The advantages of the proposed framework can be summarized as follows.

- It preserves the native resolution of small periradicular lesions, which a fixed-input resize would otherwise erase, and does so without lossy padding or lesion duplication.
- It treats severity as ordinal in both training and evaluation, so that clinically costly distant misgrades are penalized more heavily than adjacent-grade disagreements, which are more often tolerable but not uniformly so, since a slip between PAI 3 and PAI 4 can alter the follow-up interval even where it does not alter immediate treatment.
- It concentrates residual error on the immediate ordinal diagonal, with catastrophic PAI 3 versus PAI 5 confusion standing at four of 985 reference lesions.
- It provides a fair, reproducible six-generation YOLO benchmark under one protocol, giving practitioners direct evidence for architecture selection.

### 4.3. Limits of Two-Dimensional Assessment

Periapical disease is a three-dimensional process and the modality used here renders it in two. Panoramic imaging is a tomographic projection: it blurs, magnifies and displaces structures outside the focal trough and collapses a volumetric lesion onto a plane, attenuating the margin and density cues on which PAI scoring depends. Superimposition is worst in the regions most often affected, with the maxillary sinus floor obscuring or mimicking radiolucency around posterior maxillary roots and the mental foramen, incisive canal and submandibular fossa overlying premolar, incisor and posterior mandibular apices. Because loss of lamina dura continuity is the cue that separates an intact from an established periapex, any modality that renders it ambiguous preferentially produces uncertainty on the PAI 3 against PAI 4 axis, which is where both the model and, as the reader study shows, expert readers disagree most.

This raises the question of why CBCT was not used, either as a reference standard or as a second modality, and there are three reasons. The dataset is a panoramic-only public collection with no paired volumetric imaging, so a CBCT reference standard was not available for these patients and could not be obtained retrospectively. The PAI scale was defined and validated for two-dimensional periapical and panoramic radiographs, so grading against a volumetric reference would not be scoring the same construct that the scale describes. And the setting this work targets is routine panoramic examination, in which CBCT is not acquired as a matter of course and carries a higher dose and cost. The framework should therefore be read as performing panoramic-radiograph-based severity assessment and not as a substitute for three-dimensional evaluation: a lesion graded here reflects its two-dimensional radiographic appearance rather than its true volumetric extent, and a grade assigned on a panoramic image would not necessarily survive volumetric review.

### 4.4. Limitations

Several limitations apply, and we set them out in the order of how much they bound the conclusions. The reference standard describes radiographically unambiguous disease by construction, since lesions on which the three original readers disagreed were discarded before release. Performance reported here is therefore an estimate on consensus-verified lesions and is likely optimistic relative to unselected practice, where equivocal appearances are the cases for which assistance is most wanted. The collection contains no radiographs from patients free of periapical disease, since PAI 1 and PAI 2 were treated as healthy at source, so the false-positive behaviour of the framework on a healthy patient cannot be measured on this material and no claim about screening suitability is made. All radiographs come from a single centre, a single device and one acquisition protocol, which bounds population and equipment generalizability and makes external multi-centre validation necessary before any deployment. The reader study was conducted by the endodontist co-authors rather than by clinicians independent of the work, which bounds what the comparison of Section 3.8 can establish, although the readers were blinded to the reference grade and to the model output throughout. Finally, the ordinal smoothing coefficient, the severity weights and the choice of backbone were compared on the hold-out partition rather than on validation, as Section 2.9 states, so those settings are not independent of the material on which the framework is scored.

Three further limitations concern what the released data allow. The public release carries no patient identifiers, so the number of distinct patients cannot be reported and a patient-level split can neither be constructed nor verified. We treat the radiograph as the unit of analysis throughout, resample whole radiographs when forming intervals, and do not claim patient-level independence. The individual annotators’ readings are not distributed and the data descriptor reports no agreement coefficient, so the pairwise agreement of the original readers cannot be recovered, which is why the reader study of Section 2.11 was conducted. The results also rest on a single locked hold-out split, and although the repeated training runs of Section 3.2 give a measure of run-to-run variability, they share that same test set, so the variance attributable to the choice of test material is not estimated here.

Finally, two limitations concern the framework itself rather than the data. PAI 4 remains the hardest grade to separate, and its exact agreement fell in the final configuration, so the early-versus-established boundary is not resolved. And the ordinal formulation adopted here is competitive rather than optimal: as Section 3.9 shows, an established alternative attains higher severe-grade recall in isolation, and the comparison was limited to formulations that leave the detection head and its decoding unchanged, so rank-consistent heads of the CORAL family remain untested in this setting.

### 4.5. Future Work and Clinical Relevance

Future work will pursue external multi-centre validation, three-dimensional information where available and a more reliable separation of PAI 4, which Figure 18 identifies as the remaining locus of disagreement. A public panoramic benchmark carrying apical periodontitis annotations is now available, comprising 3673 radiographs and 5662 lesions [43], and although it carries no severity grades, it would allow the detection and tiling components to be tested on material from another centre without new annotation. The framework is intended as decision support under clinician oversight rather than as a replacement for the clinician, a division of responsibility that current accounts of clinical artificial-intelligence integration continue to emphasize [46], and its scope is panoramic-radiograph-based severity assessment rather than comprehensive evaluation of periapical disease. We do not claim suitability as a screening aid. That claim would require evidence on radiographs from patients with no periapical disease, which this dataset does not contain, and the present results speak only to behaviour on radiographs that carry at least one lesion.

What the study establishes is therefore technical feasibility and measured model performance rather than clinical utility. Whether a dentist reading with the framework detects more lesions, grades them more consistently, works faster or arrives at different management decisions is untested here, and each of those questions requires a prospective study in which clinicians read with and without the assistance. Until such evidence exists, the results should be read as a characterization of the model and not as a demonstration of benefit to patients.

## 5. Conclusions

This study presented a lesion-preserving tiling and ordinal severity-aware YOLO framework for the simultaneous detection and PAI grading of periapical lesions on panoramic radiographs. Mapping each radiograph onto native-resolution tiles in which every lesion stays intact improved localization for all six YOLO generations benchmarked under one identical protocol, and the gain is statistically resolvable for five of them. Adding an ordinal-aware classification loss, a high-resolution P2 head, severity class-weighting and test-time augmentation improved every headline measure at once on a locked hold-out set, with the gains concentrated on the two higher grades, although the severe-grade component of that improvement is within the resolution of this test set.

Two findings bear on how those results should be read. A reader study on the hold-out set showed that three endodontists, reading independently of one another, agreed with the reference standard and with each other less consistently than the framework did. The framework therefore reproduces the reference convention more consistently than uncalibrated expert readers, although it was trained on that convention and this is not evidence of superior clinical grading. Under label-free inference, in which no annotation is used at any point, the framework is comparable to rather than better than a detector applied to the whole down-sampled radiograph.

The contribution therefore lies in the conjunction and in the evaluation protocol rather than in any single element: a tiling rule that keeps every lesion whole at native resolution, a benchmark of six consecutive generations under one protocol, and a severity-aware objective scored with metrics that treat an ordinal scale as ordinal. The scope is panoramic-radiograph-based severity assessment rather than comprehensive three-dimensional evaluation of periapical disease, and external multi-centre validation and evaluation on radiographs from patients free of periapical disease both remain necessary before any clinical use.

## Figures and Tables

**Table 1:** Grade distribution of the dataset. Each of the three PAI grades used in this study is listed with its radiographic definition, its lesion count and its share of the 6741 annotated lesions.

Grade	Radiographic Definition	Lesions	Share (%)
PAI 3	Changes in bone structure with some mineral loss	4277	63.45
PAI 4	Periodontitis with a well-defined radiolucent area	1755	26.03
PAI 5	Severe periodontitis with exacerbating features	709	10.52

**Table 2:** The six YOLO generations benchmarked in this study, each at the medium scale, with the principal architectural change introduced by each generation. Parameter and operation counts are those published for the COCO-pretrained medium-scale models, which differ by less than 0.2 M parameters from the three-class models fine-tuned here.

Model	Year	Params (M)	GFLOPs	Principal Architectural Innovation	Inference
YOLOv8 [37]	2023	25.9	79.3	C2f backbone with an anchor-free, decoupled detection head	NMS
YOLOv9 [39]	2024	20.2	77.9	GELAN backbone with programmable gradient information (PGI)	NMS
YOLOv10 [40]	2024	16.6	64.5	Consistent dual assignments for NMS-free end-to-end detection	NMS-free
YOLO11 [41]	2024	20.1	68.5	C3k2 blocks with the C2PSA attention module	NMS
YOLO12 [42]	2025	20.2	68.1	Attention-centric backbone (area attention, R-ELAN)	NMS
YOLO26 [38]	2026	21.9	75.4	End-to-end detection head trained without NMS	NMS-free

**Table 3:** Training configuration, identical for every model and ablation.

Setting	Value	Setting	Value
GPU	RTX 4090 (24 GB)	Optimizer	MuSGD
Framework	Ultralytics 8.4.90	Initial LR (lr0, lrf)	0.01, 0.01
Precision	mixed (AMP)	Momentum	0.9
Initialization	COCO-pretrained	Weight decay	0.0005
Input size	640 × 640	Warmup epochs	3
Batch size	8	LR schedule	linear
Max. epochs	500	Loss gains (box/cls/dfl)	7.5/0.5/1.5
Early stopping	val. loss, pat. 10	HSV aug. (s, v)	0.2, 0.3
Checkpoint	best val. mAP@0.50	Geometric aug.	rot. 10°, scale 0.3, shift 0.1
Random seed	42	Flip/mosaic	h-flip 0.5/mosaic 1.0

**Table 4:** Effect of lesion-preserving tiling on each YOLO generation, reported on the hold-out test set.

Model	mAP@0.50 (No Tiling)	mAP@0.50 (Tiled)	Δ mAP@0.50	mAP (No Tiling)	mAP (Tiled)
YOLOv8m	0.460	0.543	+0.083	0.211	0.243
YOLOv9m	0.501	0.562	+0.061	0.230	0.243
YOLOv10m	0.517	0.556	+0.039	0.251	0.256
YOLO11m	0.528	0.592	+0.064	0.244	0.271
YOLO12m	0.494	0.615	+0.121	0.230	0.277
YOLO26m	0.496	0.591	+0.095	0.233	0.257

**Table 5:** Cross-generation benchmark on lesion-preserving tiles (hold-out test set, 985 lesions).

Model	mAP@0.50	mAP	Sev-wAP	QWK	Catas. (%)	PAI5 AP	PAI5 Recall
YOLOv8m	0.543	0.243	0.631	0.734	0.51	0.184	0.386
YOLOv9m	0.562	0.243	0.637	0.748	0.41	0.184	0.458
YOLOv10m	0.556	0.256	0.600	0.706	0.41	0.274	0.361
YOLO11m	0.592	0.271	0.662	0.773	0.41	0.228	0.542
YOLO12m	0.615	0.277	0.667	0.758	0.61	0.268	0.602
YOLO26m	0.591	0.257	0.650	0.720	0.71	0.238	0.482

**Table 6:** Cumulative contribution ablation on YOLO12m (hold-out test set, 985 lesions). Each row adds one component to the row above. The final row reports test-time augmentation applied to the tiled baseline instead, so that its contribution can be read separately from any interaction with the preceding components.

Configuration	mAP@0.50	mAP	Sev-wAP	QWK	Catas. (%)	PAI5 AP	PAI5 Recall
Tiled YOLO12m (baseline)	0.615	0.277	0.667	0.758	0.61	0.268	0.602
+Ordinal loss (β = 0.05)	0.609	0.279	0.691	0.753	0.41	0.249	0.506
+P2 head	0.622	0.298	0.701	0.774	0.20	0.267	0.542
+Severity weighting	0.642	0.309	0.701	0.780	0.41	0.296	0.614
+TTA (final)	0.664	0.333	0.704	0.780	0.41	0.331	0.663
Tiled YOLO12m + TTA (reference)	0.630	0.302	0.671	0.774	0.20	0.287	0.639

**Table 7:** Sensitivity of the two hyperparameters introduced in this work, reported on the hold-out test set. The upper block varies the ordinal smoothing coefficient with the P2 head held out, and the lower block varies the severity weights on top of the ordinal loss and the P2 head.

Setting	mAP@0.50	mAP	Sev-wAP	QWK	Catas. (%)	Severity MAE	PAI5 Recall
β = 0 (nominal)	0.615	0.277	0.667	0.758	0.61	0.184	0.602
β = 0.05 (adopted)	0.609	0.279	0.691	0.753	0.41	0.182	0.506
β = 0.10	0.580	0.264	0.656	0.701	1.02	0.216	0.494
w = none	0.622	0.298	0.701	0.774	0.20	0.172	0.542
w = 0.8/1.0/2.0 (adopted)	0.642	0.309	0.701	0.780	0.41	0.167	0.614
w = inverse frequency (0.53/1.28/3.17)	0.612	0.292	0.679	0.761	0.10	0.190	0.602

**Table 8:** Label-free inference on the locked test set (561 radiographs, 985 lesions). No annotation is used at any point, and window placement follows the image dimensions alone. The full-image baseline is the same detector applied to the down-sampled radiograph. The window stride, merge rule, edge handling and suppression threshold were fixed on the validation partition and applied unchanged here, and the last column counts detections above a confidence of 0.30 whose source window contains no annotated lesion. Intervals are 95% radiograph-level cluster bootstrap on the paired difference. The last column is undefined for the full-image baseline, which sees the radiograph in a single pass and therefore has no windows, so that cells and differences are left empty.

Pipeline	mAP@0.50	mAP	Sev-wAP	QWK	Catas. (%)	High-Confidence Detections from Lesion-Free Windows (%)
Full-image baseline (no tiling)	0.4937	0.2302	0.5623	0.7278	0.20	—
Label-free tiling, original training	0.3729	0.1796	0.3967	0.7895	0.30	37.5
Label-free tiling, background-augmented	0.5125	0.2203	0.5250	0.7654	0.20	9.6
Difference vs baseline	+0.019 [−0.022, +0.059]	−0.010	−0.037 [−0.065, −0.008]	+0.038 [+0.001, +0.077]	+0.00 [−0.41, +0.40]	—

**Table 9:** Reader study on a stratified sample of 249 hold-out lesions (83 per grade, comprising all PAI 5 lesions in the test set). Readers graded independently and blinded, with the lesion already localized, so the task matches what the model’s weighted kappa measures. For the three reader rows and the model row, every quantity is measured against the reference standard, and for the three pair rows it is measured between the two readers. The readers graded all 249 lesions, whereas the model row is computed on the 248 of them and localized at IoU 0.50, since its grading is conditional on localization. Intervals are 95% radiograph-level cluster bootstrapped, resampled within grade so that the stratified composition of the sample is preserved.

Grader	QWK	95% CI	Exact Agreement (%)	Severity MAE	Catastrophic (%)
Reader 1 (endodontist)	0.704	[0.628, 0.773]	66.3	0.357	2.01
Reader 2 (endodontist)	0.572	[0.493, 0.649]	56.2	0.470	3.21
Reader 3 (endodontist)	0.525	[0.441, 0.605]	49.8	0.574	7.23
Reader 1 vs. Reader 2	0.648	[0.575, 0.712]	60.6	0.410	1.61
Reader 1 vs. Reader 3	0.509	[0.417, 0.595]	49.8	0.586	8.43
Reader 2 vs. Reader 3	0.312	[0.237, 0.387]	32.9	0.843	17.27
Proposed framework	0.766	[0.700, 0.822]	73.0	0.286	1.61

**Table 10:** Comparison of ordinal formulations on the hold-out test set, all trained under the identical pipeline, partition and schedule and differing only in the classification objective. The nominal objective uses a one-hot target, the adopted method softens that target and the distance-weighted comparator leaves the target one-hot and scales each class term of the loss by one plus the squared ordinal distance from the true grade, so that a two-grade error costs two and a half times an adjacent one.

Objective	mAP@0.50	mAP	Sev-wAP	QWK	Catas. (%)	Severity MAE	PAI5 Recall
Nominal (one-hot)	0.6149	0.2775	0.6674	0.7585	0.61	0.184	0.602
Target smoothing, β = 0.05 (adopted)	0.6092	0.2788	0.6912	0.7530	0.41	0.182	0.506
Distance-weighted loss (comparator)	0.6425	0.2968	0.6830	0.7742	0.20	0.174	0.614
Full configuration (adopted + P2 + severity)	0.6418	0.3090	0.7009	0.7805	0.41	0.167	0.614

**Table 11:** Comparison with representative research studies on periapical lesion detection and severity grading. NR: not reported.

Study	Modality	Dataset	Task	Severity Handling	Reported Result
Çelik et al. [24]	Panoramic	454 lesions in 357 radiographs	Lesion detection	None	mAP = 0.953
Szabó et al. [26]	Panoramic	616 teeth	Lesion detection	None	Sensitivity = 0.78
Ba-Hattab et al. [27]	Panoramic	18,618 root areas in 713 radiographs	Root localization, then presence	None	NR
Mureșanu et al. [44]	Panoramic	1628 radiographs, 13 conditions	Multi-condition detection	None	Recall = 0.412
Fang et al. [43]	Panoramic	3673 radiographs, 5662 lesions	Lesion detection	None	AP@0.50 = 0.842
Moidu et al. [30]	Intraoral periapical	1950 radiographs, 3000 root areas	Detection with PAI 1–5	Nominal	Correct grade = 56.11%
Viet et al. [32]	Intraoral periapical	2658 radiographs	Detection with PAI 3–5	Nominal	PAI 5 correct = 91.8%
Erkal et al. [34]	Intraoral periapical	900 radiographs	Segmentation with area-based PAI	Area proxy	Accuracy = 84.5%
Saber et al. [33]	Intraoral periapical	699 radiographs	Detection with PAI 1–5	Nominal	mAP@0.50 = 0.866
Lee et al. [31]	Intraoral periapical	Not reported	Classification with PAI 1–5	Nominal, ordinal metric	QWK = 0.713
This study	Panoramic	3924 radiographs, 6741 lesions	Detection with PAI 3–5	Ordinal objective and metrics	mAP@0.50 = 0.664, QWK = 0.780

## Data Availability

Publicly available datasets were analysed in this study. The panoramic radiograph collection with periapical lesion annotations is available from Mendeley Data at https://data.mendeley.com/datasets/kx52tk2ddj/3 (accessed on 26 July 2026).

## Abbreviations

The following abbreviations are used in this manuscript.

AMP	automatic mixed precision
AP	average precision
CBCT	cone-beam computed tomography
CNN	convolutional neural network
COCO	common objects in context
GELAN	generalized efficient layer aggregation network
GFLOPs	giga-floating-point operations per forward pass
GPU	graphics processing unit
HSV	hue, saturation, value
IoU	intersection over union
MAE	mean absolute error
mAP	mean average precision
MuSGD	muon and stochastic gradient descent optimizer
NMS	non-maximum suppression
PAI	periapical index
PGI	programmable gradient information
QWK	quadratic weighted kappa
Sev-wAP	severity-weighted average precision
TTA	test-time augmentation
YOLO	you only look once
